# The Impact of Drying Technologies and Thermal Processing on the Nutritional Profiles, Flavor Fingerprints, and Taste Attributes of Giant Freshwater Prawn (*Macrobrachium rosenbergii*)

**DOI:** 10.3390/foods15183354

**Published:** 2026-09-21

**Authors:** Yanna Cui, Yafang Shen, Jiangqi Wang, Qiuyue Hu, Yuanyuan Li, Fei Jia, Guijie Hao

**Affiliations:** 1 Key Laboratory of Healthy Freshwater Aquaculture, Ministry of Agriculture and Rural Affairs, Huzhou Key Laboratory of Aquatic Product Quality Improvement and Processing Technology, Zhejiang Institute of Freshwater Fisheries, Huzhou 313001, China; 15967216452@163.com (Y.C.); shenyafang1994@126.com (Y.S.); jiangqiwang@163.com (J.W.); chenyang10140021@163.com (Q.H.); 2 College of Food Science and Engineering, Ocean University of China, Qingdao 266000, China; liyuanyuan01032022@163.com (Y.L.); feijia@ouc.edu.cn (F.J.)

**Keywords:** giant freshwater prawn, drying methods, thermal processing, flavor remodeling, nutritional components, electronic nose, electronic tongue

## Abstract

This study systematically investigated the effects of vacuum freeze-drying (VFD) and hot-air drying (HAD), combined with cooking and seasoning, on the nutritional composition, volatile flavor fingerprints, and non-volatile taste profiles of giant freshwater prawn (*Macrobrachium rosenbergii*) meat. Proximate composition, amino acid and fatty acid profiles, electronic nose, electronic tongue, and GC-MS were employed to characterize the quality evolution across nine treatment groups. Results demonstrated that drying significantly concentrated proteins, lipids, and amino acids, yet the retention efficiency was highly process-dependent. In cooked prawn, VFD exhibited superior preservation of heat-labile amino acids and polyunsaturated fatty acids, whereas HAD induced greater fatty acid retention in raw material. Notably, cystine was identified exclusively in unseasoned cooked groups, suggesting heat-induced oxidative dimerization of cysteine, while its absence in seasoned samples indicated interference from Maillard-driven reducing conditions. Flavor analysis revealed divergent mechanisms: VFD primarily exerted a physical concentration effect, preserving endogenous fatty acid methyl esters, while HAD triggered intensive thermochemical reactions, generating nitrogenous compounds (e.g., N, N-dimethylmethanamine) and roasted markers. Electronic tongue analysis indicated that VFD-cooked prawn achieved an optimal umami-richness balance, whereas seasoning imposed a dominant salty-acidic mask over inherent sweetness. Collectively, VFD is recommended for premium ready-to-eat products prioritizing nutritional integrity and authentic flavor, while HAD suits products targeting intense roasted sensory characteristics. This study provides a mechanistic framework and practical guidance for drying technology selection in aquatic product processing.

## 1. Introduction

Giant freshwater prawn (*Macrobrachium rosenbergii*) stands as one of the most commercially valuable aquatic products globally, highly prized for its high protein content, abundance of essential amino acids, and unique flavor profile [1]. However, due to its high moisture content and nutrient-rich composition, giant freshwater prawn meat is highly susceptible to rapid spoilage caused by microbial growth and enzymatic activity [2]. To extend shelf life and facilitate distribution, various processing methods have been employed, among which drying remains a traditional and effective preservation technique [3]. Meat floss, a popular dried meat product characterized by its loose, fibrous texture and savory taste, involves multiple production stages, including cooking, shredding, seasoning, and drying [4]. The quality of the final product, particularly its nutritional value and aroma characteristics, is profoundly influenced by the specific drying method employed during manufacturing [5].

Different drying technologies, such as hot-air drying (HAD), vacuum freeze drying (VFD), microwave drying, and solar drying, exert distinct effects on the physicochemical properties of the food matrix [6]. While hot-air drying is cost-effective and widely used, it often involves high temperatures that can lead to significant degradation of nutrients, lipid oxidation, and the formation of undesirable off-flavors [7]. Studies indicate that thermal treatment alters protein structures, reduces the bioavailability of essential amino acids, and promotes the Maillard reaction; while this contributes to desirable roasted notes, uncontrolled conditions may also generate harmful compounds [8]. In contrast, vacuum freeze drying operates under low-temperature and vacuum conditions, effectively preserving the material’s original morphology, nutritional components, and volatile flavor compounds [9]. Reports suggest that compared to conventional thermal drying, this method retains higher quality proteins and carbohydrates in shrimp products [10].

The aroma profile of shrimp is a critical determinant of consumer acceptance, composed of a complex mixture of Volatile Organic Compounds (VOCs) generated during processing [11]. Key aroma-active compounds in shrimp include aldehydes, ketones, alcohols, and sulfur-containing substances, which originate from lipid oxidation, amino acid degradation, and the Maillard reaction [12]. The type of drying method significantly impacts the composition and concentration of these volatile compounds. Vacuum freeze drying better preserves the original constituents of dried shrimp, whereas hot-air drying facilitates the formation of new taste compounds [13]. Furthermore, VFD is most effective in retaining heat-sensitive components such as astaxanthin, albeit at a higher cost; conversely, while HAD may cause some isomerization and degradation of astaxanthin, it maintains high biological activity with greater efficiency under optimized conditions [14]. Recent research indicates that the interaction between processing conditions and drying technologies determines the final sensory attributes, with certain methods enhancing sweetness and caramel notes, while others may exacerbate fishy or rancid odors [15].

Despite extensive research on the drying of whole shrimp or shrimp powder, the research object is limited to *Litopenaeus vannamei*. *Litopenaeus vannamei* and *Macrobrachium rosenbergii* have a great difference in meat quality. The crude fat content, unsaturated fatty acid content, and essential amino acid index of *Macrobrachium rosenbergii* were significantly higher than those of *Litopenaeus vannamei* [16]. There remains a lack of comprehensive studies regarding the dynamic changes in nutrients and aroma compounds across different processing stages of giant freshwater prawn floss under varying drying conditions [17]. Understanding how specific drying methods influence the evolution of quality parameters from raw material to the final floss product is crucial for optimizing processing technologies [18,19]. This study aims to investigate the effects of two distinct drying methods on the nutritional content and volatile aroma profiles of giant freshwater prawn floss at various processing stages. By comparing the retention of proteins, lipids, and key flavor compounds, this research seeks to provide a theoretical basis and practical guidance for producing high-quality giant freshwater prawn floss with superior nutritional and sensory characteristics.

## 2. Materials and Methods

### 2.1. Samples

Giant freshwater prawns (*Macrobrachium rosenbergii*) were procured from a commercial supplier (Along Aquatic Products Store, Huzhou, Zhejiang, China) on 25 August 2025. The specimens had been originally harvested via seine netting. The average prawn weight was 35.5 g.

According to different drying processes, the samples were divided into the following nine categories: A (raw prawn meat), B (VFD raw prawn meat), C (HAD raw prawn meat), D (cooked prawn meat without seasoning), E (VFD cooked prawn meat without seasoning), F (HAD cooked prawn meat without seasoning), TD (cooked prawn meat with seasoning), TE (VFD cooked prawn meat with seasoning), TF (HAD cooked prawn meat with seasoning).

The composition of the seasoning (5 kg shelled prawns, with an average prawn meat yield of 36.3%): 2500 g raw smoke, 900 g rock sugar, 20 g sweet leaves, 20 g star anise, 30 g cinnamon, 50 g plum, 120 g ginger, 120 g green onion, 450 g lemon, 2500 g carved wine, 4 L pure water.

VFD method: A total of 5 kg of prawns (the sample size was determined according to the required experimental shrimp meat) were processed by boiling for 3–5 min to obtain the meat. All procedures were conducted in accordance with ethical guidelines to minimize animal suffering. The prawn meat was soaked in a seasoning solution for flavoring. The seasoned prawn meat was pre-cooled to 0–4 °C. The pre-cooled prawn meat underwent vacuum freeze-drying for 21 h. The samples were freeze-dried using a vacuum freeze dryer (LABCONCO, Kansas City, MO, USA). During the lyophilization process, the vacuum pressure was maintained at 0.035 mbar, and the condenser (collector) temperature was set to −84 °C. The vacuum freeze-dried prawn meat was crushed to produce prawn meat floss [20].

HAD method: The method to obtain prawn meat was the same as VFD. The prawn meat was soaked in a seasoning solution for flavoring. The soaked prawn meat was dried at 70 °C for 21 h.

Prawn meat floss was a dehydrated, fibrous, fluffy processed product obtained from boiled prawn meat (*Macrobrachium rosenbergii*) after seasoning, drying (via vacuum freeze-drying or hot-air drying), and mechanical crushing. It was characterized by low water activity, high protein content, and a savory flavor, commonly used as a seasoning or ready-to-eat snack.

There was no significant difference in the weight of prawns soaked in each hot-air drying and vacuum freeze drying process.

### 2.2. Analysis of Basic Nutrients

The protein content (GB 5009.5-2016 first method [21]), fat content (GB 5009.6-2016 first method [22]), carbohydrate content (GB 28050-2011 [23]), moisture content (GB 5009.3-2016 first method [24]), ash content (GB 5009.4-2016 first method [25]), sodium content (GB 5009.91-2017 fourth method [26]), total fatty acid content (GB 5009.168-2016 first method [27]), the sum of the amino acids determined using the procedures employed in this study (see Section 2.3) of samples A, B, C, D, E, F, TD, TE and TF were identified. The differences between the samples were compared. Each sample was analyzed 4 times repeatedly.

### 2.3. Analysis of Amino Acid Content

Tryptophan Determination: The sample was crushed to pass through a 40-mesh sieve and thoroughly mixed. An accurately weighed 0.2 g portion of the homogenized sample was transferred into a 35 mL hydrolysis tube. Using a 10 mL pipette, 10 mL of 4 mol/L sodium hydroxide solution was added. The tube was flushed with high-purity nitrogen, immediately sealed tightly with the screw cap, and placed in an electric thermostatic drying oven at 110 °C for hydrolysis for 24 h. After hydrolysis, the tube was removed and allowed to cool to room temperature. The hydrolysate was quantitatively transferred to a 50 mL volumetric flask. The hydrolysis tube was rinsed several times with sodium acetate buffer solution, and 7.5 mL of 6 mol/L hydrochloric acid was added to the combined rinsate for neutralization. After thorough mixing and cooling to room temperature, the solution was made up to volume with sodium acetate buffer solution and mixed well. The resulting solution was centrifuged, and the supernatant was filtered through an organic membrane prior to liquid chromatography analysis.

Liquid chromatography conditions were as follows: column, Waters Symmetry C18 (4.6 × 250 mm, 4.6 μm); column temperature, 40 °C; detection wavelength, 280 nm; injection volume, 10 μL; flow rate, 1.2 mL/min; run time, 16 min; mobile phase A, acetonitrile (5%); mobile phase B, acetic acid–sodium acetate buffer (95%): 2.5 g sodium acetate, 1.5 mL triethylamine, and 1.17 mL glacial acetic acid dissolved in 1 L of purified water.

Other Amino Acids (excluding tryptophan): An accurately weighed amount of sample was transferred into a hydrolysis tube such that the protein content in the sample was within the range of 10–20 mg (approximately 100 mg for Sample D, and approximately 25 mg for Samples E and F). Then, 15 mL of 6 mol/L hydrochloric acid solution (containing 0.1% phenol) was added. The hydrolysis tube was placed in a freezing mixture and frozen for 3–5 min, then evacuated under vacuum and flushed with nitrogen. The tube was finally sealed or tightly capped under a nitrogen atmosphere. The sealed hydrolysis tube was placed in an oven at 110 °C for hydrolysis for 22 h. After hydrolysis, the tube was removed and allowed to cool to room temperature. The hydrolysis tube was opened, and the hydrolysate was filtered into a 50 mL volumetric flask. The hydrolysis tube was rinsed several times with small portions of water, and the washings were combined into the same 50 mL volumetric flask. The solution was finally made up to volume with water, mixed thoroughly, and shaken. An aliquot of 1.0 mL of the filtrate was accurately transferred into a 15 mL test tube and evaporated to dryness under a stream of nitrogen using a test tube concentrator at 50 °C. The dried residue was redissolved in water and evaporated to dryness again. The resulting residue was reconstituted with an appropriate volume of pH 2.2 sodium citrate buffer solution, mixed well, and filtered through a membrane. The filtrate was then analyzed using a Scion Artemis 6000C amino acid analyzer (Scion Instruments, Livingston, Scotland, United Kingdom).

### 2.4. Analysis of Fatty Acid Content

Fatty acid composition was determined following the method described in GB 5009.168-2016, with minor modifications. An appropriate amount of homogenized prawn sample (approximately 0.5–1.0 g, accurately weighed to 0.001 g) was transferred into a screw-capped hydrolysis tube, and a known volume of internal standard solution (glyceryl triundecanoate, C11:0 triglyceride) was added. For samples with visible fat content, direct saponification and methylation were performed. For samples requiring complete lipid recovery, the following extraction procedure was applied: the sample was hydrolyzed under appropriate conditions, followed by the addition of ethanol with thorough mixing. Acetic acid and petroleum ether were then added sequentially, and the mixture was subjected to liquid–liquid extraction three consecutive times. The upper organic layers from each extraction were combined and evaporated to dryness. The resulting lipid residue was subjected to alkaline saponification and methylation to convert fatty acids into their corresponding fatty acid methyl esters (FAMEs). The resulting FAMEs were collected and redissolved in n-hexane for subsequent GC analysis.

Fatty acid methyl esters were analyzed using an Agilent 7820A gas chromatograph (Agilent Technologies, Santa Clara, CA, USA) equipped with a flame ionization detector (FID). Chromatographic separation was performed on an Agilent HP-88 capillary column (100 m × 0.25 mm i.d., 0.20 μm film thickness). The injection port and detector temperatures were set at 270 °C and 280 °C, respectively. Helium (purity ≥ 99.999%) was used as the carrier gas at a constant flow rate of 1.0 mL/min. Samples were injected in split mode with a split ratio of 30:1. The column oven temperature program was as follows: initial temperature 100 °C held for 13 min; ramped to 180 °C at 10 °C/min and held for 6 min; ramped to 200 °C at 1 °C/min and held for 12 min; ramped to 230 °C at 10 °C/min and held for 8 min.

Fatty acid peaks were identified by comparing retention times with those of authentic FAME standards (Supelco 37-component FAME mix, Sigma-Aldrich, St. Louis, MO, USA). Quantification was performed using the internal standard calibration method (glyceryl triundecanoate, C11:0 triglyceride). Results were expressed as grams of each fatty acid per 100 g of dry matter (g/100 g dry weight). All samples were analyzed in quadruplicate (n = 4), and results are reported as mean ± standard deviation (SD).

### 2.5. Analysis of Electronic Nose

The sample was broken into mud, and 2 g of the sample was taken and placed in a 50 mL centrifuge tube. Each sample was taken in triplicate, sealed with a sealing membrane, and placed for 30 min. After enrichment, the test was performed. The injection needle was directly inserted into a sealed centrifuge tube containing the sample using the direct headspace suction method, and the determination was carried out using the German PEN3 electronic nose((AIRSENSE Analytics GmbH, Schwerin, Germany). Determination conditions: The carrier gas for the electronic nose was purified air, and the analysis was performed at room temperature. Sampling time was 1 s/group; the self-cleaning time of the sensor was 60 s. The sample injection time was 5 s; the injection flow rate was 400 mL min^−1^. The analysis sampling time was 80 s. The data from 69–71 s were taken as the results for summary and analysis. Ten sensors and corresponding sensitive substances are shown in Table 1. Each sample was analyzed 9 times repeatedly.

### 2.6. Analysis of Electronic Tongue

Six sensors (C00, AE1, CA0, CT0, AAE, GL1) and three standard electrodes were used. The sensors were tested after activation and correction. The matching information between the sensor and the taste value is shown in Table 2.

Sample 1: 20 was smashed with pure water, centrifuged and filtered, and then identified by the SA402B electronic tongue. Balance: The sensor was washed in the cleaning solution for 90 s, the reference solution for 120 s, and then another reference solution for 120 s. The sensor was zeroed at the equilibrium position for 30 s. Test: The test time was 30 s, and the first taste value was recorded; after cleaning with the reference solution for 3 s, the sensor was inserted into a new reference solution to test the aftertaste for 30 s. The food five-flavor sensors C00, AE1, CA0, CT0, AAE and the bitterness sensors ANO and BTO were tested 4 times. The average value of the last three times was taken as the test result after removing the first cycle. The sweetness sensor GL1 was tested 5 times, and the average value of the middle 3 tests was taken as the test result after removing the first and last cycles. Each sample was analyzed 3 times repeatedly.

### 2.7. Analysis of HS-SPME-GC-MS

Sample Pretreatment: Each sample (1–2 g, accurately weighed to 0.001 g) was transferred into a 20 mL headspace vial compatible with the solid-phase microextraction (SPME) autosampler. Subsequently, 100 µL of a 0.1% (*v*/*v*) cyclohexanone internal standard working solution was added, and the mixture was thoroughly vortexed for 30 s to ensure homogenization. The vials were then placed in the heating module of the GC–MS system and equilibrated at 70 °C for 10 min under gentle agitation. After equilibration, the SPME fiber (80 µm DVB/carbon/PDMS, Agilent China Ltd., Beijing, China) was exposed to the headspace for 40 min to allow adsorption of volatile compounds. Finally, the fiber was retracted and immediately introduced into the GC injection port for thermal desorption at 250 °C for 5 min.

Instrumental Conditions: Chromatographic separation was performed using an Agilent 8890 gas chromatograph coupled with an Agilent 7000E triple quadrupole mass spectrometer (Agilent Technologies, Santa Clara, CA, USA). The injection port temperature was maintained at 250 °C, and all injections were performed in splitless mode. Helium (purity ≥ 99.999%) was used as the carrier gas at a constant flow rate of 1.0 mL/min. The analytes were separated on an HP-5MS UI capillary column (30 m × 0.25 mm i.d., 0.25 µm film thickness; Agilent). The solvent delay time was set to 1.0 min. The column oven temperature program was as follows: initial temperature 40 °C held for 4 min, increased to 90 °C at a rate of 3 °C/min and held for 3 min, then raised to 100 °C at 1 °C/min and held for 1 min, followed by an increase to 120 °C at 3 °C/min with a 1 min hold, and finally ramped to 250 °C at 6 °C/min and held for 3 min.

The mass spectrometer was operated in electron ionization (EI) mode at an electron energy of 70 eV. The temperatures of the ion source, quadrupole, and transfer line were set to 200 °C, 150 °C, and 250 °C, respectively. Full-scan mass spectra were acquired over a mass-to-charge ratio (*m*/*z*) range of 35–500. All samples were analyzed in triplicate (n = 3) to ensure data reproducibility.

To evaluate potential matrix effects arising from the internal standard, blank samples (prepared without the addition of cyclohexanone) were analyzed in parallel with spiked samples (containing 100 µL of 0.1% cyclohexanone) under identical instrumental conditions to determine whether any target compounds co-eluted with or were transformed from the internal standard. This validation was performed for all sample groups (raw, cooked, and seasoned) to ensure the reliability of volatile compound identification.

### 2.8. Statistical Analysis

All experiments were conducted with at least three independent biological replicates per treatment group (n ≥ 3), with each replicate derived from separately processed prawn samples to ensure true biological variability was captured. Analytical determinations for proximate composition, amino acids, and fatty acids were performed in quadruplicate (n = 4) per biological replicate, while electronic tongue and GC-MS analyses were performed in triplicate (n = 3), and electronic nose analysis in nonuplicate (n = 9). Results are expressed as mean ± standard deviation (SD).

Statistical analyses were performed using SPSS Statistics (version 19.0, IBM Corp., Armonk, NY, USA). Prior to parametric testing, the normality of data distribution was assessed using the Shapiro–Wilk test (*p* > 0.05 indicating normality), and homogeneity of variances was verified using Levene’s test (*p* > 0.05 indicating equal variances). For datasets satisfying both assumptions, one-way analysis of variance (ANOVA) followed by Tukey’s honestly significant difference (HSD) post hoc test was employed for pairwise multiple comparisons. For datasets that violated the assumption of normality or homogeneity of variances, the non-parametric Kruskal–Wallis test was applied, followed by Dunn’s post hoc test with Bonferroni correction for multiple comparisons to control the family-wise error rate.

A significance threshold of α = 0.05 was set for all statistical tests, and differences were considered statistically significant when *p* < 0.05. Different superscript letters in tables and figures denote significant differences among groups.

For multivariate datasets generated by electronic nose and electronic tongue analyses, principal component analysis (PCA) was performed using OriginPro 2026b (OriginLab Corporation, Northampton, MA, USA) to visualize sample clustering and identify key discriminating variables, for E-nose and E-tongue; and SA402B Taste Sensing System software Taste Analysis Application V1.1/V1.2, Insent Inc., Atsugi, Japan, for E-tongue) to visualize sample clustering and identify key discriminating variables. The cumulative variance contribution rates of the first two principal components (PC1 and PC2) were reported to evaluate the adequacy of the dimensionality reduction. All PCA models were validated using full cross-validation (leave-one-out) to assess model robustness. Prior to PCA, data were auto-scaled (mean-centered and divided by the standard deviation) to eliminate scale effects among variables.

Graphical visualization, including radar charts, biplots, circular heatmaps, and chord diagrams, was generated using OriginPro 2026b (OriginLab Corporation, Northampton, MA, USA). The reproducibility of all instrumental analyses was verified by calculating the relative standard deviation (RSD) for replicated runs, with acceptance criteria set at RSD < 15% for GC-MS and E-tongue data, and RSD < 10% for E-nose sensor responses. Outliers were identified using the Grubbs’ test (α = 0.05) and were excluded only when analytical or instrumental errors were unequivocally confirmed; otherwise, all valid data points were retained in the final analysis.

## 3. Results and Analysis

### 3.1. Proximate Composition (Expressed on Dry Matter Basis, Except Moisture)

All nutritional parameters—including protein, fat, carbohydrate, ash, sodium, total fatty acids, and total amino acids—are reported on a dry matter basis (g/100 g dry weight). Moisture content is presented separately as wet-basis percentage to reflect processing-induced water removal efficiency.

#### 3.1.1. Protein Content

Protein values ranged from 71.18 ± 0.12 g/100 g (TE) to 89.27 ± 0.10 g/100 g (C). Among raw samples, C (oven-dried raw, 89.27) was significantly higher than A (fresh raw, 86.32) and B (freeze-dried raw, 83.75) (*p* < 0.05), indicating that oven-drying may cause a relative concentration of proteinaceous components even on a dry basis. For cooked non-seasoned groups, D (85.29), E (85.91), and F (85.61) were statistically similar (*p* > 0.05) and also comparable to A, suggesting that cooking alone does not significantly alter protein content on a dry matter basis. Notably, all seasoned samples (TD 75.08, TE 71.18, TF 74.76) were significantly lower than their unseasoned counterparts (D/E/F) (*p* < 0.05), with TE showing the minimum.

#### 3.1.2. Fat and Total Fatty Acids

Fat content increased from 3.20 ± 0.31 (A) to 11.18 ± 0.42 g/100 g (TD). Raw B (4.26) and C (4.65) were not significantly different (*p* > 0.05), but both exceeded A. Cooked non-seasoned Samples D–F (8.54–8.98) formed a homogeneous cluster (*p* > 0.05) and were significantly higher than raw groups, implying that cooking promotes lipid concentration or retention even after dry-weight normalization, possibly due to protein denaturation and structural changes that reduce fat loss during processing. Seasoned TD showed the highest fat content (11.18), whereas TE (6.70) and TF (7.00) were significantly lower than TD but higher than raw samples, reflecting differential seasoning absorption and fat-binding capacities among drying methods. Total fatty acids followed an almost identical trend, with values from 2.97 (A) to 8.44 (TD). All groups were significantly different except E vs. F (*p* > 0.05). The strong correlation between fat and total fatty acids indicates that most lipids are present as fatty acids, and processing effects on fat are primarily physical rather than compositional.

#### 3.1.3. Carbohydrate Content

Carbohydrate content exhibited extremely high variability, ranging from 0.08 ± 0.01 g/100 g (C) to 14.72 ± 0.57 g/100 g (TE). In the absence of seasoning, freeze-dried raw prawn (B, 6.13 ± 0.86 g/100 g) showed significantly higher carbohydrate content than fresh raw prawn (A, 4.10 ± 0.44 g/100 g) (*p* < 0.05). Similarly, freeze-dried cooked prawn (E, 0.65 ± 0.06 g/100 g) exhibited a significantly higher value than its undried counterpart (D, 1.66 ± 0.17 g/100 g) (*p* < 0.05). This phenomenon can be explained by the fact that during freeze-drying, low-temperature sublimation rapidly removes moisture, creating a porous structure within the prawn matrix and leading to shrinkage of the dry matter skeleton, thereby increasing the relative proportion of carbohydrates on a dry weight basis. Meanwhile, freeze-drying causes minimal degradation loss of carbohydrates, thus resulting in an apparent accumulation effect on a dry matter basis.

However, the effects of oven drying differed markedly. Oven-dried raw prawn (C, 0.08 ± 0.01 g/100 g) and oven-dried cooked prawn (F, 0.75 ± 0.05 g/100 g) were not significantly different from each other (*p* > 0.05), and both remained at extremely low levels. This is primarily attributed to the elevated temperatures employed during oven drying (typically 50–70 °C or higher), which accelerate carbohydrate degradation and consumption. On the one hand, high temperatures promote the Maillard reaction between reducing sugars and free amino acids in prawn meat, consuming substantial amounts of sugars. On the other hand, prolonged heating may induce caramelization or thermal decomposition of certain oligosaccharides, further reducing carbohydrate content [28]. Additionally, the slow dehydration process during oven drying may cause sugars to migrate to the surface along with moisture, where they undergo thermal degradation losses, resulting in far lower carbohydrate levels on a dry weight basis compared with freeze-dried samples [29].

Upon seasoning addition, carbohydrate content increased dramatically regardless of drying method: TD (seasoned cooked, undried) reached 6.25 ± 0.10 g/100 g, TF (seasoned oven-dried cooked) reached 10.60 ± 0.11 g/100 g, and TE (seasoned freeze-dried cooked) reached as high as 14.72 ± 0.57 g/100 g, all significantly higher than their corresponding unseasoned counterparts (D/E/F) (*p* < 0.05). This substantial increase is unequivocally attributable to the exogenous addition of carbohydrate-based ingredients in the seasoning formulation (e.g., sucrose, maltodextrin, and starch). Among them, the seasoned freeze-dried group (TE) exhibited the highest carbohydrate content, which resulted from both exogenous sugar addition and the concentration effect of freeze-drying on dry matter. All three seasoned groups were significantly higher than their unseasoned counterparts, indicating that seasoning is the predominant factor determining the carbohydrate content of prawn products, far outweighing the effects of processing methods per se.

#### 3.1.4. Ash and Sodium Content

Ash content ranged from 4.51 ± 0.09 (D) to 7.64 ± 0.10 g/100 g (TF). Raw Samples A (6.38), B (5.86), and C (6.00) were all significantly different (*p* < 0.05), suggesting that drying methods affect mineral retention or concentration differently. Cooked non-seasoned D–F showed the lowest ash values (4.51–4.66) and were statistically homogeneous (*p* > 0.05), implying that cooking may leach certain minerals. Seasoned samples TD, TE, and TF exhibited the highest ash contents (7.50–7.64), with TF significantly exceeding TD and TE (*p* < 0.05), confirming that freeze-drying better preserves the original ash proportion of the sample. Sodium content, although extremely low in all non-seasoned groups (0.30–0.40 mg/100 g, *p* > 0.05), surged dramatically in seasoned products: TD 1.63, TE 1.99, and TF 1.84 mg/100 g (all significantly different from each other and from non-seasoned groups, *p* < 0.05). This unequivocally identifies added salt as the primary sodium source, and the variation among seasoned groups reflects different salt incorporation efficiencies during processing.

#### 3.1.5. Total Amino Acid Content

Total amino acids ranged from 62.32 ± 2.47 (A) to 91.10 ± 1.84 g/100 g (E). Raw B (85.88), C (82.67), and cooked D (83.73) were statistically similar (*p* > 0.05), while E (91.10) and F (90.31) were significantly higher than all other groups. This suggests that combined cooking and drying—especially freeze-drying after cooking—may enhance the extractability or measurement of amino acids without actual net synthesis. Seasoned Samples TD, TE, and TF (70.97–71.23) were not significantly different from each other (*p* > 0.05) but were markedly lower than their non-seasoned counterparts, again demonstrating a dilution effect from seasoning matrices rather than protein/amino acid degradation.

#### 3.1.6. Moisture Content (Wet Basis, Not Included in Dry-Matter Calculations)

Moisture content (g/100 g wet sample) ranged from 5.36 ± 0.09 (E) to 76.56 ± 0.30 (A), and all groups were significantly different from each other (*p* < 0.05). Fresh raw A had the highest moisture, while all dried products (B, C, E, F, TE, TF) showed substantially lower values (5.36–21.93). Notably, D and TD retained high moisture comparable to fresh samples. These data confirm that drying processes (freeze-drying and oven-drying) are highly effective in moisture removal, and that seasoning does not interfere with drying efficiency. Importantly, because all nutritional components are expressed on a dry matter basis, moisture differences do not confound comparisons among groups.

#### 3.1.7. Summary of Processing Effects

Cooking alone (D vs. A) increased fat and fatty acids but decreased ash on a dry basis. The protein content remained stable, and the total amino acid content increased. Drying alone (B/C vs. A) produced variable effects: freeze-drying had a better retention effect on amino acids and carbohydrates, while drying significantly increased protein, ash, and total fatty acids. Seasoning was the dominant factor, markedly elevating sodium, ash, and carbohydrates, while diluting protein, amino acids, and (in some cases) total fatty acids.For further details, see Table 3.

### 3.2. Amino Acid Composition (Dry Matter Basis)

The amino acid profiles of prawn samples subjected to different processing conditions are presented in Table 4. A total of 18 amino acids were identified. All amino acid contents are expressed on a dry matter basis (g/100 g dry weight). Significant differences were observed among treatment groups for most amino acids (*p* < 0.05), reflecting the substantial impact of processing methods on the amino acid profile of prawn meat.

#### 3.2.1. Effects of Drying on Amino Acid Content in Raw Prawn (A vs. B vs. C)

Compared with fresh raw prawn (A), both freeze-drying (B) and oven-drying (C) significantly increased the contents of most amino acids (*p* < 0.05). Lysine and arginine exhibited the most pronounced increases. Lysine content increased from 4.69 ± 0.24 g/100 g in A to 7.67 ± 0.23 g/100 g in B and 7.12 ± 0.06 g/100 g in C (*p* < 0.05). Arginine content increased from 3.62 ± 0.10 g/100 g in A to 8.43 ± 0.78 g/100 g in B and 8.21 ± 0.05 g/100 g in C (*p* < 0.05). Similar increasing trends were observed for Asp, Glu, and Leu, with values in dried samples being significantly higher than those in fresh samples (*p* < 0.05) or showing increasing tendencies. These results suggest that even on a dry matter basis, both VFD and HAD effectively increase the contents of most amino acids in prawn.

#### 3.2.2. Effects of Drying on Amino Acid Content in Cooked Prawn (D vs. E vs. F)

In cooked prawn, the effects of different drying methods on amino acid contents followed similar trends to those observed in raw prawn. Compared with cooked prawn (D), freeze-dried cooked prawn (E) and oven-dried cooked prawn (F) generally exhibited increased contents of most amino acids. Lysine increased from 8.27 ± 0.27 g/100 g in D to 9.02 ± 0.16 g/100 g in E and 9.02 ± 0.36 g/100 g in F (*p* < 0.05). Tryptophan content in E (9.41 ± 0.14 g/100 g) was significantly lower than in D (9.52 ± 0.06 g/100 g) and F (10.66 ± 0.07 g/100 g).

#### 3.2.3. Effects of Cooking and Seasoning on Amino Acid Content (D, E, F vs. TD, TE, TF)

Comparison between cooked prawn (D, E, F) and seasoned cooked prawn (TD, TE, TF) revealed that seasoning exerted significant and differential effects on the amino acid profile. Negative effects (significant decreases): Seasoning generally caused significant reductions in amino acid content. Leu, Tyr, and Phe showed no significant differences between any cooked groups (D, E, F) and their seasoned counterparts (TD, TE, TF) (*p* > 0.05). Cystine exhibited a unique distribution pattern. It was not identified (0.00 g/100 g) in raw groups (A, B, C) or in any seasoned groups (TD, TE, TF), but was consistently identified in cooked unseasoned groups D (0.34 ± 0.12), E (0.35 ± 0.03), and F (0.44 ± 0.11) (*p* < 0.05).

#### 3.2.4. Major Trends and Key Findings in Amino Acid Profiles

**Figure 1:**
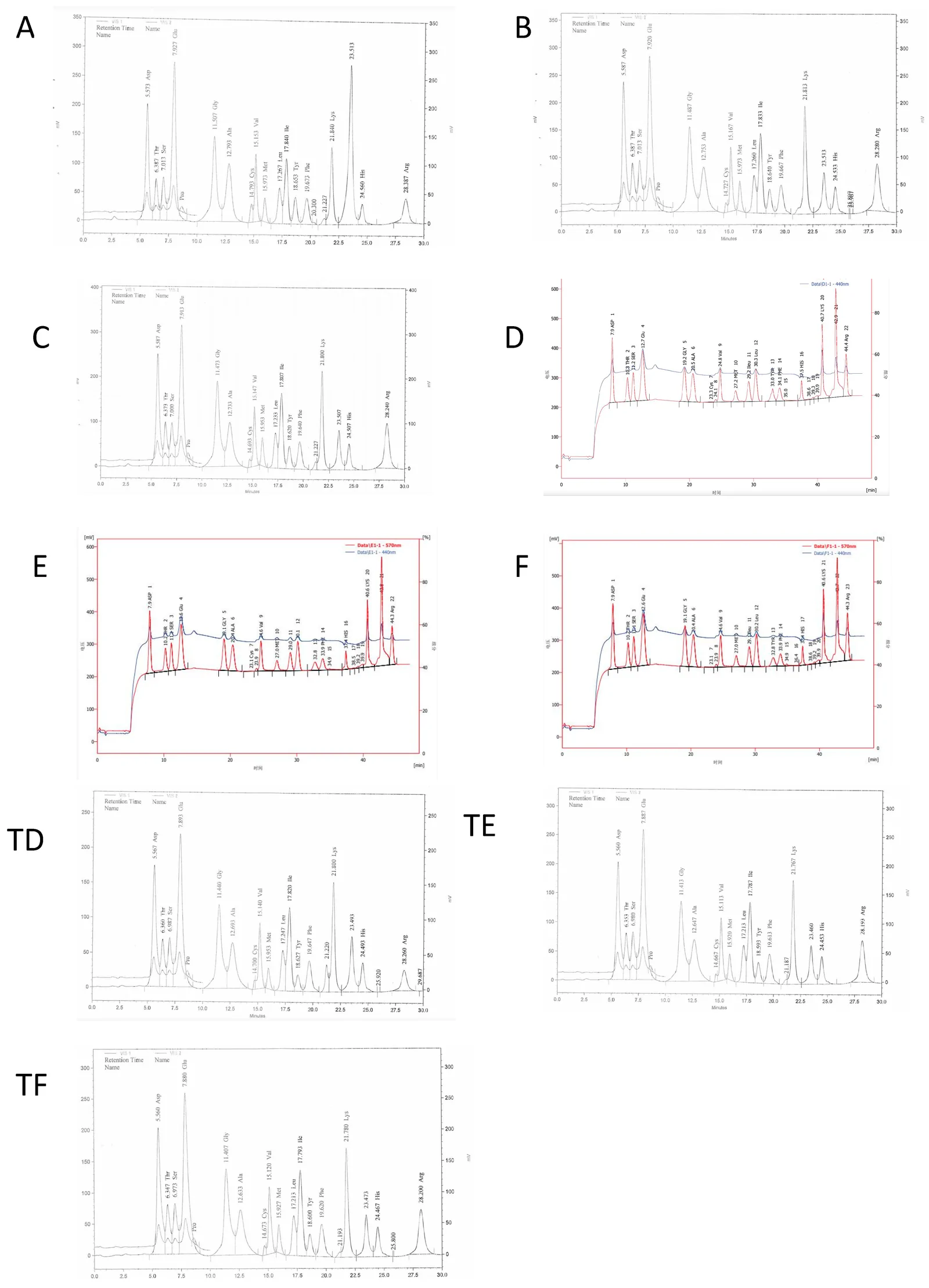
Amino acid spectrum under different processing treatments (A, B, C, D, E, F, TD, TE, TF).

Based on the comprehensive amino acid data, the following major trends and key findings can be summarized. Drying effect: Both VFD and HAD significantly increased the contents of most amino acids per unit dry matter in both raw and cooked prawn (Except for Trp). The enrichment effect of VFD on amino acids was better than HAD. Cooking effect: Cooking significantly increased most amino acid contents but significantly decreased Tyr and Trp (*p* < 0.05). Seasoning effect: Most of the amino acids were significantly reduced in the seasoning group. Amino acid retention characteristics: Cystine was only identified in the unseasoned cooked group, but not in all fresh and seasoned groups, suggesting that reducing sugars or salts in seasonings may interfere with the stability or detection of cystine. Tryptophan was well retained in raw and seasoned freeze-dried samples, but significantly lost in unseasoned cooked samples, indicating that the choice of processing method and seasoning formula was crucial to the retention of tryptophan.The amino acid spectrum is shown in Figure 1.

### 3.3. Fatty Acid Composition (Dry Matter Basis)

The fatty acid profiles of prawn samples subjected to different processing conditions are presented in Table 5. A total of 14 major fatty acids were identified, including 5 saturated fatty acids (SFAs: C14:0, C15:0, C16:0, C17:0, C18:0), 4 monounsaturated fatty acids (MUFAs: C16:1, C18:1, C20:1), and 5 polyunsaturated fatty acids (PUFAs: C18:2, C18:3, C20:2, C20:4, EPA, DHA). All fatty acid contents are expressed on a dry matter basis (g/100 g dry weight). Significant differences were observed among treatment groups for most fatty acids (*p* < 0.05), reflecting the substantial impact of processing methods on the lipid profile of prawn meat.

#### 3.3.1. Effects of Drying on Fatty Acid Content in Raw Prawn (A vs. B vs. C)

Compared with fresh raw prawn (A), both freeze-drying (B) and oven-drying (C) increased the contents of most fatty acids. Significant decreases were observed for C20:1, which decreased from 0.018 ± 0.001 g/100 g in A to 0.014 ± 0.001 g/100 g in B (*p* < 0.05), and for C16:1, which decreased from 0.091 ± 0.007 g/100 g in A to 0.058 ± 0.004 g/100 g in B and C 0.058 ± 0.001 g/100 g (*p* < 0.05). No significant differences (*p* > 0.05) were found among A, B, and C for EPA (C20:5n-3), C18:0, C14:0, C15:0, C18:3, C18:2, and C18:1, indicating that drying of raw prawn did not substantially alter the contents of these fatty acids on a dry matter basis.

#### 3.3.2. Effects of Drying on Fatty Acid Content in Cooked Prawn (D vs. E vs. F)

In cooked prawn, the effects of VFD (E) and HAD (F) compared with cooked undried prawn (D) showed that approximately 50% of the identified fatty acids exhibited no significant differences, while the remaining 50% showed significant increases. These results indicate that drying applied after cooking can further increase the concentration of certain fatty acids per unit dry matter.

#### 3.3.3. Effects of Cooking and Seasoning on Fatty Acid Content (D, E, F vs. TD, TE, TF)

Comparison between cooked prawn (D, E, F) and seasoned cooked prawn (TD, TE, TF) revealed that Direct seasoning of cooked prawn (TD) significantly increased the contents of most fatty acids compared with unseasoned cooked prawn (D). In contrast, seasoned and subsequently dried Samples (TE and TF) generally exhibited significantly lower fatty acid contents compared with their unseasoned dried counterparts (E and F). C15:0 exhibited a unique distribution pattern. It was not identified (0.00 g/100 g) in raw groups (A, B, C) or in any seasoned groups (TD, TE, TF), but was consistently identified in cooked unseasoned groups D (0.014 ± 0.016), E (0.036 ± 0.003), and F (0.039 ± 0.001) (*p* < 0.05). This suggests that the cooking process may induce the formation or release of this odd-chain fatty acid, while the addition of seasonings may interfere with its detection or stability.

#### 3.3.4. Major Trends and Key Findings in Fatty Acid Profiles

Based on the comprehensive fatty acid data, the following major trends and key findings can be summarized:

Drying effect: The effect of drying on fatty acid content varied depending on the raw material state. For raw prawn, both VFD and HAD significantly increased the contents of certain fatty acids such as DHA. For cooked prawn, drying further increased approximately 50% of the identified fatty acids, while the remaining 50% showed no significant changes.

Cooking effect: Cooking was a critical step influencing the fatty acid profile. It not only significantly increased the contents of most fatty acids, but also induced the formation or release of C15:0, which was not identified in any unheated samples.

Seasoning effect: Seasoning exerted a dual effect on fatty acid content. Direct seasoning of cooked prawn (TD) increased most fatty acid contents, likely due to the contribution of exogenous lipids from seasoning ingredients. However, when seasoning was followed by drying (TE, TF), most fatty acid contents decreased significantly compared with unseasoned dried samples (E, F).

Major fatty acid composition: C16:0 (palmitic acid), C18:1 (oleic acid), and C18:2 (linoleic acid) were consistently the most abundant fatty acids across all treatment groups.

### 3.4. Analysis of Non-Volatile Taste Profiles of Prawn Meat Using Electronic Tongue Technology

To elucidate the effects of different processing methods (drying, cooking, and seasoning) on the overall taste profile of shrimp meat, principal component analysis (PCA) was performed on the electronic tongue data. In all subplots, the first two principal components (PC1 and PC2) exhibited high cumulative variance contribution rates, indicating that these two dimensions effectively captured the flavor differences among samples. The direction and length of the loading vectors represent the contribution of each taste indicator to the principal components and their degree of association with the samples.

#### 3.4.1. Overall Analysis of Flavor Characteristics Based on PCA

Across all six subplots, the cumulative variance explained by PC1 and PC2 ranged from 84.3% to 91.7%, confirming that a two-dimensional PCA solution adequately represented the electronic tongue response patterns. The loading plots revealed that basic tastes—particularly bitterness, sourness, sweetness, saltiness, and umami—together with mouthfeel attributes (astringency, richness, and aftertaste) constituted the primary axes of discrimination. In general, PC1 predominantly separated raw/untreated samples from dried/processed ones, while PC2 further distinguished samples based on seasoning status and drying method. The following sections systematically examine the flavor evolution at each processing stage.

#### 3.4.2. Effect of Raw Material State and Drying Method on Flavor Profiles

##### Flavor Evolution of Raw Prawn Meat and Its Processed Products (Figure 2A)

For the raw prawn group, PC1 explained 73.1% of the total variance and served as the primary discriminating dimension. Sample A (raw prawn): Positioned on the negative side of PC1, Sample A was closely associated with the loading vectors of bitterness, sourness, and sweetness. This suggests that fresh raw prawn possesses a distinctive baseline flavor profile, likely originating from the balanced interplay of free amino acids and nucleotides in the muscle tissue. Moreover, the absence of heat treatment means that proteins remain undenatured, preserving a relatively primitive biochemical flavor signature. Sample B (freeze-dried raw prawn) and Sample C (oven-dried raw prawn): Both samples shifted markedly towards the positive side of PC1. Sample B exhibited strong positive correlations with saltiness, umami, richness, and aftertaste-B, indicating that the freeze-drying process effectively concentrated the taste-active compounds. Sample C, in contrast, showed stronger associations with aftertaste-A and astringency, suggesting that oven-drying may promote different chemical transformations (e.g., Maillard reaction products or lipid oxidation derivatives) that contribute to a more astringent and lingering mouthfeel. From the cluster distribution, Sample B largely overlapped with Samples A and C, with only moderate discrimination among the three. This implies that while drying significantly altered the taste intensity, the overall flavor orientation of raw-derived products remained partially comparable.

**Figure 2:**
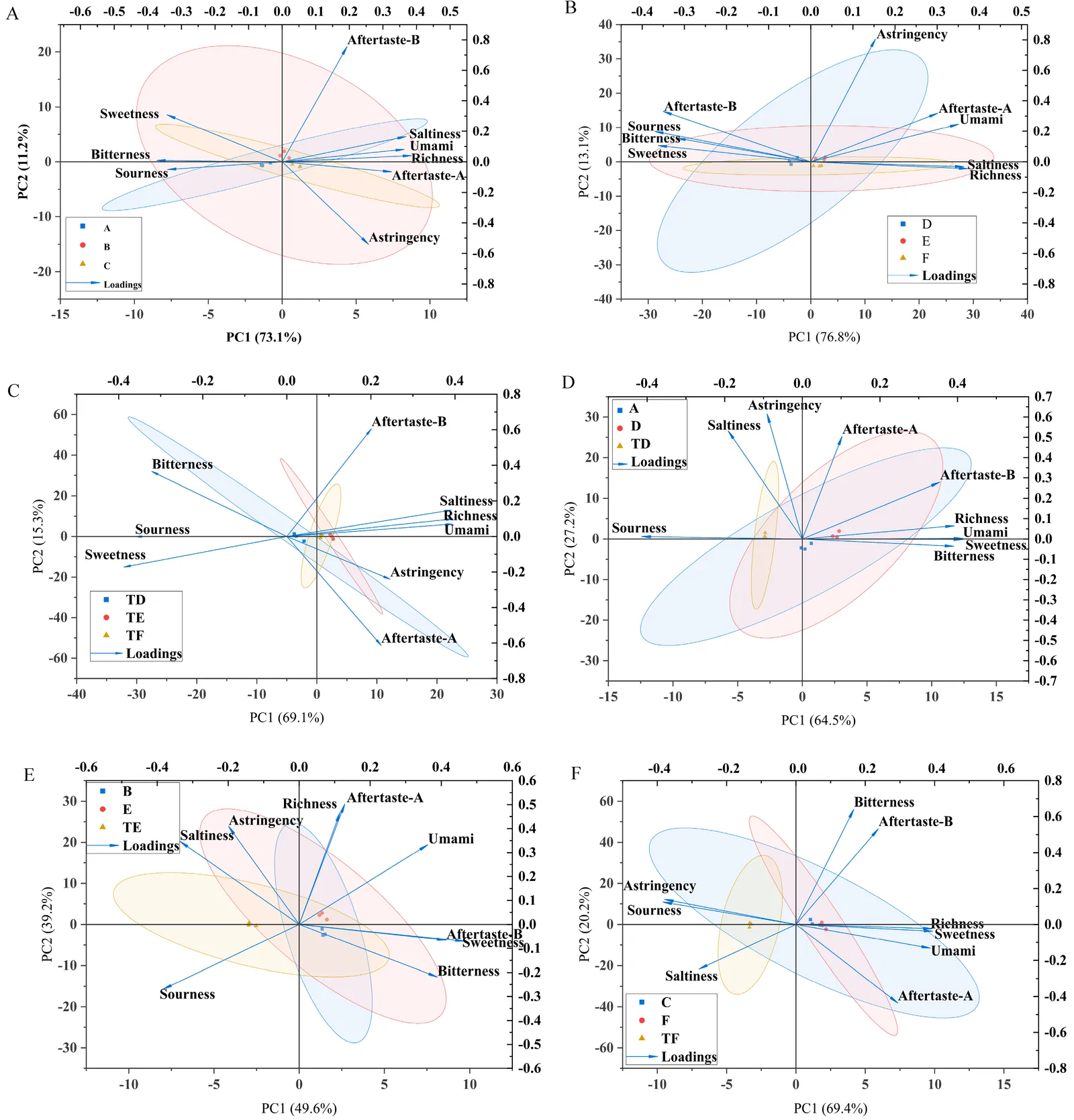
The comparison of different treatment samples in the biplot analysis of electronic tongue test results.The shaded areas represent the 95% confidence ellipses for each sample group, and different colors correspond to different groups. The lines represent the loading vectors of the original variables. (**A**) Comparison of processing differences in raw prawn meat; (**B**) Comparison of processing differences in cooked prawn meat; (**C**) Comparison of processing differences in seasoned cooked prawn meat; (**D**) Comparison of processing differences in undried prawn meat; (**E**) Comparison of processing differences in VFD prawn meat; (**F**) Comparison of processing differences in HAD prawn meat.

##### Flavor Evolution of Cooked Prawn Meat and Its Processed Products (Figure 2B)

In the cooked prawn group, PC1 accounted for 76.8% of the variance, indicating a strong primary differentiation axis. Sample D (cooked prawn): Located on the negative side of PC1, Sample D was strongly correlated with aftertaste-B, bitterness, sourness, and sweetness. This profile differs notably from that of raw prawn (Sample A in Figure 1A), suggesting that the simple cooking process (heat treatment) induced Maillard reactions and protein denaturation, which restructured the taste matrix and generated cooked-meat-specific flavor notes. Sample E (freeze-dried cooked prawn) and Sample F (oven-dried cooked prawn): Both dried samples were distributed on the positive side of PC1. Sample E was primarily driven by astringency, aftertaste-A, and umami, whereas Sample F was more associated with saltiness and richness. This distinction implies that the two drying methods, when applied to pre-cooked material, lead to divergent concentration and/or generation of taste compounds—freeze-drying preserving or enhancing umami-related nucleotides, while oven-drying intensifies salty and kokumi-like sensations. Notably, Sample E completely encompassed Sample F in the biplot, indicating no clear discrimination between the two dried cooked products. This suggests that once the prawn is cooked, the subsequent drying method exerts a relatively minor influence on the overall taste profile compared to the cooking step itself.

#### 3.4.3. Reshaping of Flavor Profiles by Seasoning Treatment

##### Analysis of Seasoned Cooked Prawn and Its Processed Products (Figure 2C)

Following the introduction of seasoning, PC1 explained 69.1% of the variance.

Sample TD (seasoned cooked prawn): Positioned on the negative side of PC1, Sample TD was mainly influenced by bitterness, sourness, and sweetness. This may be attributed to the inherent flavors of the seasoning formula (e.g., spices, acidulants, or sweeteners) or to the masking effect of these added ingredients, which partially suppressed the umami notes originally present in cooked prawn. Sample TE (seasoned freeze-dried cooked prawn) and Sample TF (seasoned oven-dried cooked prawn): Both samples were located on the positive side of PC1 and clustered closely around the loading vectors of saltiness, umami, and richness. This indicates that regardless of the drying method, the combination of seasoning and dehydration markedly enhanced the salty-umami-rich taste profile. The high umami scores observed for TE and TF can be attributed to the synergistic effect of added flavor enhancers (e.g., monosodium glutamate or nucleotides from seasoning blends) and the endogenous amino acids released from prawn muscle proteolysis, further amplified by the concentration effect during drying.

In contrast to the cooked-only groups, Samples TD, TE, and TF exhibited strong discrimination from one another, confirming that seasoning not only intensifies taste but also diversifies the flavor space.

#### 3.4.4. Horizontal Comparative Analysis of Key Processing Nodes

To further clarify the specific contribution of each processing step, we conducted pairwise and cross-group comparisons at critical nodes.

##### Cooking Effect: Raw vs. Cooked vs. Seasoned Cooked (Figure 2D: A vs. D vs. TD)

Samples A (raw), D (cooked), and TD (seasoned cooked) exhibited distinct positioning in the biplot. Sample A (raw): Located on the negative side of PC1, close to the bitterness vector, reflecting the unprocessed biochemical state. Sample D (cooked): Shifted towards the positive side of PC1, showing stronger associations with aftertaste-A, aftertaste-B, richness, sweetness, and umami. This confirms that heat treatment is a critical step in transforming the flavor structure—reducing raw bitterness while enhancing cooked-meat savoriness and lingering mouthfeel. Sample TD (seasoned cooked): Positioned in the upper region of PC2, predominantly influenced by astringency, saltiness, and sourness. This deviation indicates that the seasoning marinade significantly altered the basic taste balance of cooked prawn, introducing acidic and salty notes that may originate from lemon juice or salt in the formulation. From the cluster perspective, Samples A, D, and TD showed considerable overlap, with no highly significant discrimination. This suggests that the mere addition of seasoning to cooked prawn, without subsequent drying, does not create a sufficiently distinct taste space to fully separate it from unseasoned cooked or raw samples.

##### Effect of Freeze-Drying: Raw vs. Cooked vs. Seasoned Cooked Freeze-Dried Products (Figure 2E: B vs. E vs. TE)

Sample B (raw freeze-dried): Positioned on the positive side of PC1, primarily influenced by aftertaste-B, sweetness, and bitterness. Sample E (cooked freeze-dried): Also on the positive side of PC1, but shifted closer to the umami, richness, and aftertaste-A vectors. This displacement suggests that cooking prior to freeze-drying better preserves or transforms the precursors of umami substances (e.g., inosine monophosphate and free glutamic acid), yielding a more savory final product compared to raw freeze-dried prawn. Sample TE (seasoned cooked freeze-dried): Located on the negative side of PC1, driven mainly by sourness, astringency and saltiness. This stark contrast indicates that the specific seasoning formula—possibly containing acidic components or high salt levels—overwhelmed the intrinsic sweet-umami notes of prawn, dominating the taste profile.

Notably, Samples B, E, and TE exhibited substantial overlap in the biplot, with no clear differentiation. This implies that under freeze-drying conditions, the addition of seasoning does not produce a flavor profile that is distinctly separable from its unseasoned counterparts, likely due to the mild dehydration process that limits intense flavor transformation.

##### Effect of Oven-Drying: Raw vs. Cooked vs. Seasoned Cooked Oven-Dried Products (Figure 2F: C vs. F vs. TF)

Sample C (raw oven-dried) and Sample F (cooked oven-dried): Both samples were located on the positive side of PC1 and were strongly driven by bitterness, aftertaste-B, umami, sweetness, richness, and aftertaste-A. This co-localization suggests that oven-drying, regardless of whether applied to raw or cooked material, produces a broadly similar taste orientation characterized by concentrated savory and sweet notes with pronounced bitterness and lingering aftertaste. Sample TF (seasoned cooked oven-dried): In sharp contrast, sample TF deviated significantly to the negative side of PC1 and was strongly associated with astringency, sourness, and saltiness. This dramatic reversal indicates that the combination of oven-drying and seasoning induces distinct chemical interactions—possibly enhanced Maillard reaction products or the surface enrichment of salt and organic acids during the drying process—that generate a fundamentally different taste profile, dominated by acidic and astringent sensations rather than the typical savory-sweet balance. Regarding cluster distribution, Sample C almost completely encompassed Samples F and TF, while the overlap between F and TF was relatively limited. This suggests that when oven-drying is applied, the presence or absence of seasoning creates a more pronounced flavor divergence than the raw/cooked status of the starting material.

In summary, the electronic tongue PCA biplots revealed a consistent hierarchy of processing effects on prawn flavor:

1. Drying (both freeze-drying and oven-drying) significantly concentrated taste compounds and shifted samples towards positive PC1 values, enhancing umami, richness, and salty notes.

2. Cooking transformed the raw flavor matrix, reducing bitterness and increasing cooked-meat savoriness and aftertaste complexity.

3. Seasoning exerted a dominant effect that could either amplify (in dried products) or mask (in non-dried products) the intrinsic prawn taste, depending on the drying method applied.

4. The drying method played a secondary but still important role, with freeze-drying favoring umami retention and oven-drying intensifying bitterness and astringency, especially when combined with seasoning.

5. Clustering patterns indicated that raw-based products (A, B, C) and cooked-based products (D, E, F) formed partially overlapping groups, while seasoned products (TD, TE, TF) showed the highest intra-group diversity, underscoring the profound reshaping effect of seasoning on taste profiles.

#### 3.4.5. Electronic Tongue Taste Profile Analysis

**Figure 3:**
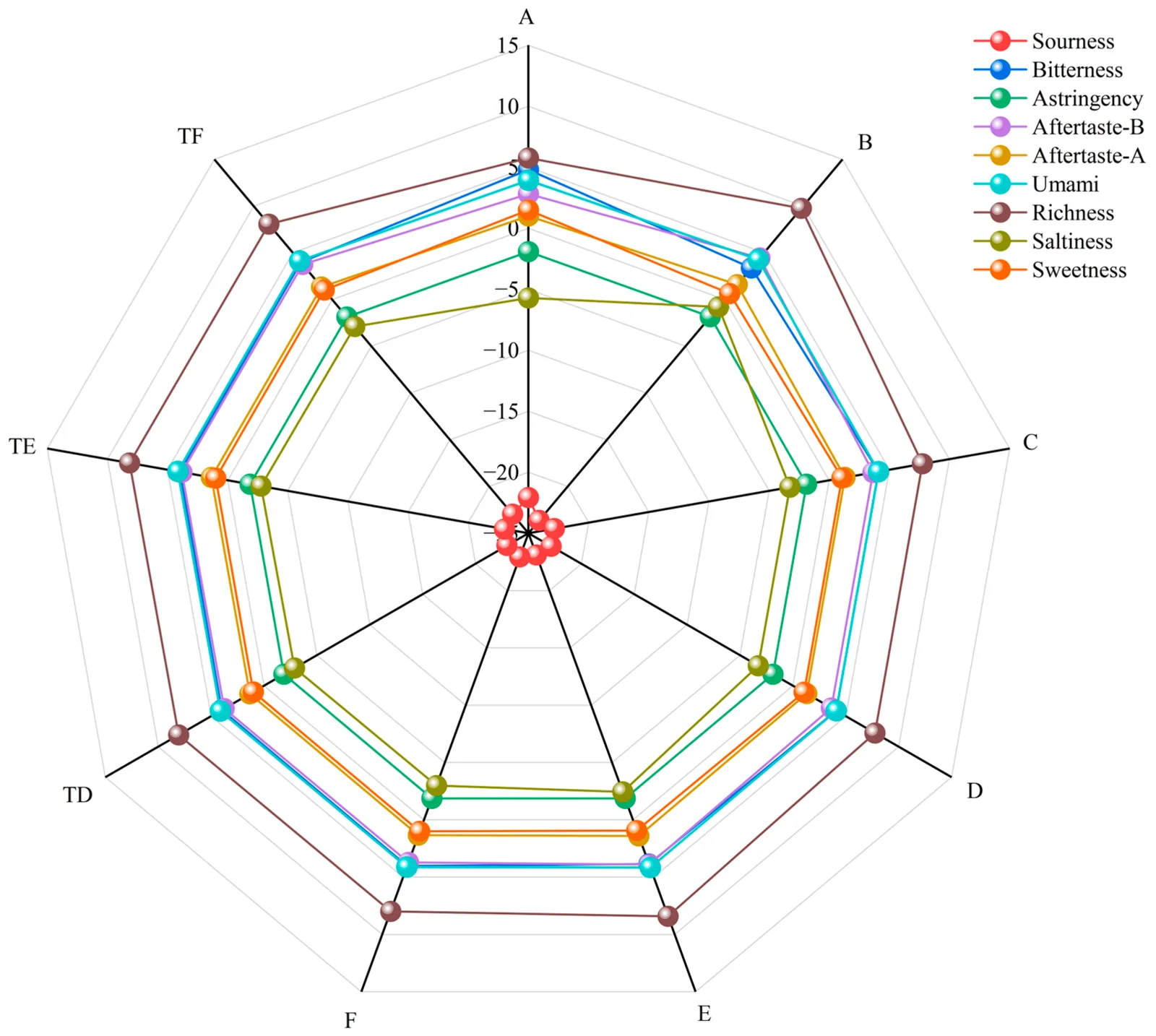
The comparison of different treatment samples of radar map in electronic tongue test results.

To comprehensively evaluate the impact of different processing conditions on the taste characteristics of prawn products, an electronic tongue (E-tongue) system was utilized to analyze the taste fingerprints of nine sample groups (A, B, C, D, E, F, TD, TE, and TF). The results are presented in the radar chart shown in Figure 3, which illustrates the profiles of each group across nine taste dimensions: Sourness, Bitterness, Astringency, Aftertaste-B, Aftertaste-A, Umami, Richness, Saltiness, and Sweetness.

Among the nine sample groups, fresh raw prawn (A) and freeze-dried raw prawn (B) exhibited distinctly contrasting taste characteristics. Group A was characterized by a significantly higher level of Bitterness compared to all other samples, while its Saltiness, Aftertaste-A, Aftertaste-B, and Richness were remarkably lower than the rest. In contrast, Group B exhibited significantly higher values in Saltiness, Aftertaste-A, Aftertaste-B, and Richness compared to the other samples; however, its Sweetness and Bitterness were lower than those of the other groups. This indicates that the purely physical freeze-drying process created a distinct taste profile, which drastically enhanced the release of saltiness and aftertastes while failing to generate the sweetness typically derived from thermal treatments, and simultaneously mitigated the innate bitterness of raw prawn. Apart from groups A and B, the other seven sample groups exhibited almost identical taste characteristics.

### 3.5. Principal Component Analysis (PCA) Results Based on Electronic Nose Technology

Principal component analysis (PCA) was employed to investigate the effects of different processing techniques (fresh, freeze-drying, hot-air drying) and seasoning treatments on the volatile flavor compounds of prawn. Figure 2A–F show the PCA biplots for the different sample groups. In these plots, the blue arrows represent the loading vectors of the electronic nose sensors, with their lengths and directions reflecting the contribution rates and correlations of the sensors to the separation of the principal components.

#### 3.5.1. Analysis of Processing Differences in Raw Prawn (A, B, and C)

**Figure 4:**
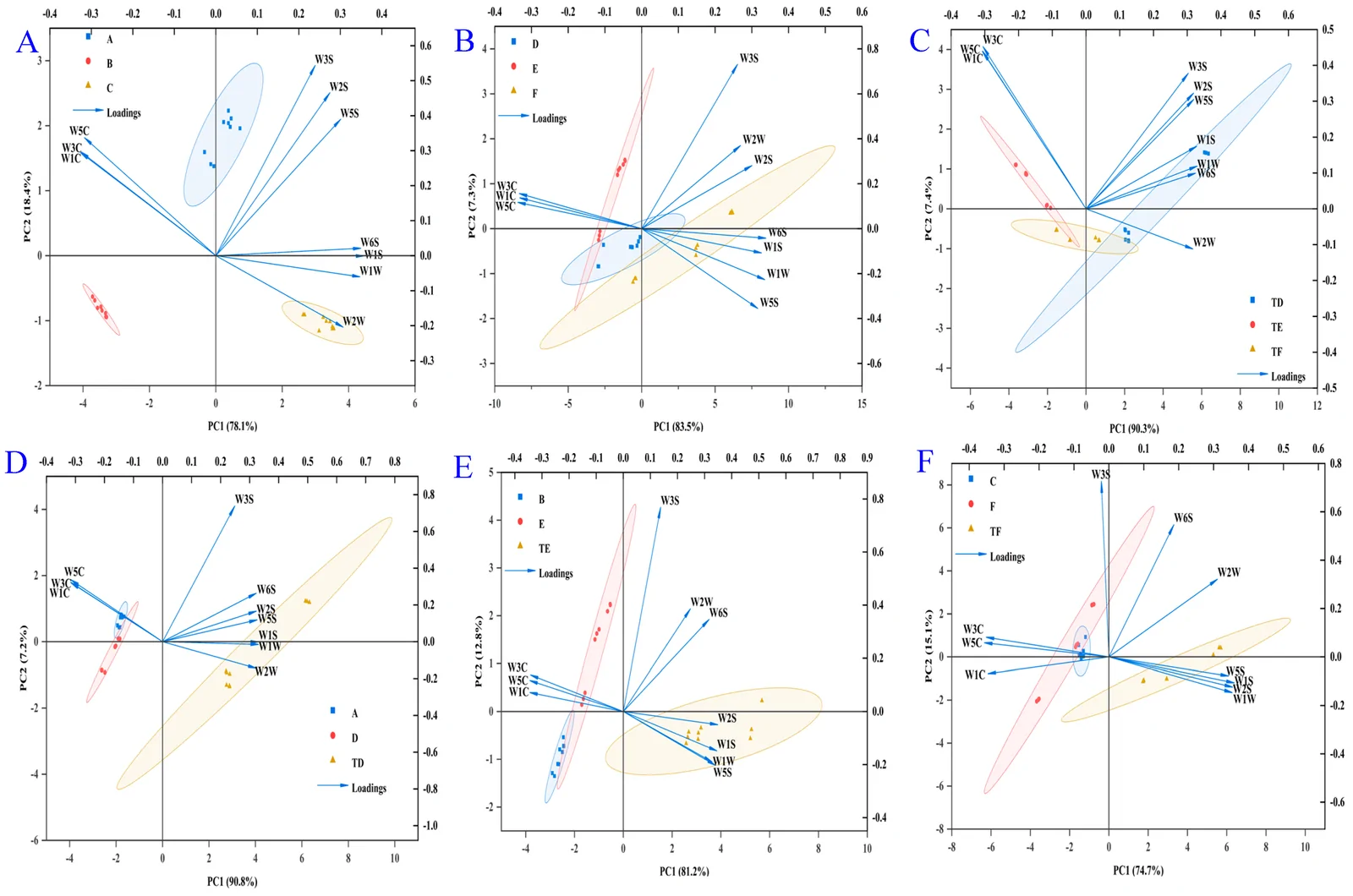
The comparison of different treatment samples of PCA in electronic nose test results. (**A**) Comparison of processing differences in raw prawn meat; (**B**) Comparison of processing differences in cooked prawn meat; (**C**) Comparison of processing differences in seasoned cooked prawn meat; (**D**) Comparison of processing differences in undried prawn meat; (**E**) Comparison of processing differences in VFD prawn meat; (**F**) Comparison of processing differences in HAD prawn meat.

As shown in Figure 4A, raw prawn (A), freeze-dried raw prawn (B), and hot-air dried raw prawn (C) exhibited distinct spatial separation along PC1 (78.1%) and PC2 (18.4%). Raw prawn (A) was primarily distributed in the first and second quadrants, with its flavor characteristics strongly positively correlated with multiple sensors on the right and upper sections, mainly involving W1C (aromatic compounds), W3C (ammonia and aromatic molecules), W5C (olefins, aromatics, and polar molecules), W3S (alkanes and aliphatics), W2S (alcohols and some aromatic compounds), and W5S (nitrogen oxides). This suggests that fresh prawn retain a relatively comprehensive and abundant profile of original volatile compounds. Freeze-dried raw prawn (B) was independently located in the third quadrant, and its confidence ellipse did not show a strong correlation with any sensor’s loading vector. This implies that the freeze-drying process may have caused significant changes or losses in volatile compounds, resulting in a fingerprint profile distinctly different from the other processed samples. Hot-air dried raw prawn (C) was situated in the fourth quadrant, showing a significant correlation with the bottom sensors W1S (alkanes), W1W (sulfur compounds), and W2W (aromatic compounds and organosulfur compounds). This may be attributed to lipid oxidation and thermal degradation reactions during hot-air drying, which generated specific alkanes and sulfur-containing compounds.

#### 3.5.2. Influence of Cooking and Drying Processes on Cooked Prawn (D, E, and F)

Figure 4B illustrates the distribution of cooked prawn (D), freeze-dried cooked prawn (E), and hot-air dried cooked prawn (F). The heating process is critical for flavor development in meat products, and the differences in drying methods continued to exert a dominant influence. Freeze-dried cooked prawn (E) was intensely skewed toward the negative semi-axis of PC1 (predominantly in the second quadrant), with a highly elongated confidence ellipse, indicating a highly uniform recognition signal after freeze-drying. Its loadings were strongly correlated with W1C, W3C, and W5C (aromatic compounds, ammonia, and polar molecules), demonstrating that the freeze-drying process effectively locked in the key nitrogen-containing and aromatic volatile compounds generated during cooking. Hot-air dried cooked prawn (F) was located in the first, third, and fourth quadrants with a broad confidence ellipse, reflecting significant intra-batch variability in volatile compound distribution due to hot-air drying, and it was correlated with multiple sensor signals. Notably, cooked prawn (D) was located in the third quadrant and did not exhibit significant loading correlations with any sensor. This indicates that cooked prawn, without undergoing any drying process, had a flavor profile that neither leaned toward the fidelity of freeze-drying nor possessed the typical thermal flavor products generated by high-temperature drying, thus representing a transitional state.

#### 3.5.3. Restructuring Effect of Seasoning on Prawn Flavor Characteristics

Figure 4C (TD, TE, TF) and subsequent figures reveal the masking or synergistic effects of the seasoning process on the substrate flavors. Seasoned freeze-dried cooked prawn (TE) remained anchored in the second quadrant, similar to the aforementioned freeze-dried cooked prawn (E), once again confirming the high retention of aromatic and ammonia-based molecules (W1C, W3C, W5C) by the freeze-drying process. The seasoning did not disrupt the flavor constraints imposed by the physical structure created by freeze-drying but instead caused TE to shift slightly higher along PC2. Seasoned hot-air dried prawn (TD/TF) exhibited a pronounced stretching toward the positive direction of PC1, and this trend was highly correlated with the W2W (aromatic compounds and organosulfur compounds) sensor. This is highly likely due to complex Maillard reactions and sulfur amino acid degradation occurring during the high-temperature drying of seasonings (e.g., sugars and spices), which generated abundant sulfur-containing heterocyclic compounds and aromatic compounds, thus profoundly reshaping the prawn flavor fingerprint.

#### 3.5.4. Evaluation of Process-Oriented Comprehensive Flavor Profiles

Figure 4E (comparison of B, E, TE) and Figure 4F (comparison of C, F, TF) present the process matrices centered on freeze-drying and hot-air drying, respectively, further elucidating the decisive role of drying methods combined with subsequent thermal processing/seasoning on the flavor profile of prawn. In the freeze-drying-related process matrix (Figure 4E), freeze-dried cooked prawn (E) and seasoned freeze-dried cooked prawn (TE) exhibited significant differences in confidence ellipse span and loading correlations. The confidence ellipse of E spanned the first, second, and third quadrants, predominantly located in the negative PC1 and positive PC2 regions. Its flavor characteristics were highly correlated with multiple sensors located at the top, including W2W (aromatic and organosulfur compounds), W6S (hydrides), W3S (alkanes and aliphatics), W3C (ammonia and aromatic molecules), W5C (olefins, aromatics, and polar molecules), and W1C (aromatic compounds). This indicates that the cooking process activated a broad network of volatile compounds within the freeze-dried matrix. Conversely, the seasoned TE group showed a marked displacement of its confidence ellipse, spanning the first, third, and fourth quadrants, extending toward the negative PC2 and positive PC1 directions. Its correlations primarily shifted to the bottom sensors: W1W (sulfur compounds), W2S (alcohols and some aromatic compounds), W1S (alkanes), and W5S (nitrogen oxides). This suggests that the seasoning process, through the addition of sugars and spices, successfully suppressed some of the original aromatic/olefinic characteristics while simultaneously constructing a new flavor system dominated by alcohols, alkanes, and specific sulfur compounds.

Furthermore, in the freeze-drying matrix, the unheated and unseasoned freeze-dried raw prawn (B) was concentrated independently in the third quadrant, with its confidence ellipse not showing significant loading correlations with any sensor. This indicates that the purely physical dehydration process of freeze-drying, lacking subsequent cooking reactions or Maillard reactions in the substrate, failed to trigger further flavor transformation, leaving its volatile characteristics in a blank transitional region of the profile.

In the hot-air drying-related process matrix (Figure 4F), seasoned hot-air dried prawn (TF) exhibited a trend highly similar to TE, with its confidence ellipse also spanning the first, third, and fourth quadrants, and its correlated sensors similarly concentrated at the bottom (W1W, W2S, W1S, W5S). This demonstrates that, regardless of whether the initial substrate was freeze-dried or hot-air dried, the seasoning process (particularly when accompanied by high-temperature treatment) produces a remarkably similar deviation effect—shifting the center of gravity of the flavor profile from a “structure-retaining type” (high melting point, cyclic) towards a “thermally induced type” (alcohols, short-chain alkanes, nitrogen oxides). In contrast, the unseasoned hot-air dried raw prawn (C) was located in the second and third quadrants, retaining its original drying characteristics highly correlated with W1C, W5C, and W3C (aromatic compounds and polar small molecules).

Combining Figure 4E,F, it is evident that “cooking” and “seasoning” are two key technological nodes that break the simple physical drying state of raw prawn. Cooking extends the volatile profiles of freeze-dried or hot-air dried matrices toward multiple aromatic and alkane-type sensors (E/F characteristics), while seasoning further forces the center of gravity of the flavor profile downward (TE/TF characteristics), forming a new pattern dominated by alcohols, alkanes, and sulfur compounds. This fully demonstrates that, in the flavor construction of prawn products, the seasoning step plays a decisive modifying role in their final sensory profile.

#### 3.5.5. Analysis of Electronic Nose Sensor Response Profiles

**Figure 5:**
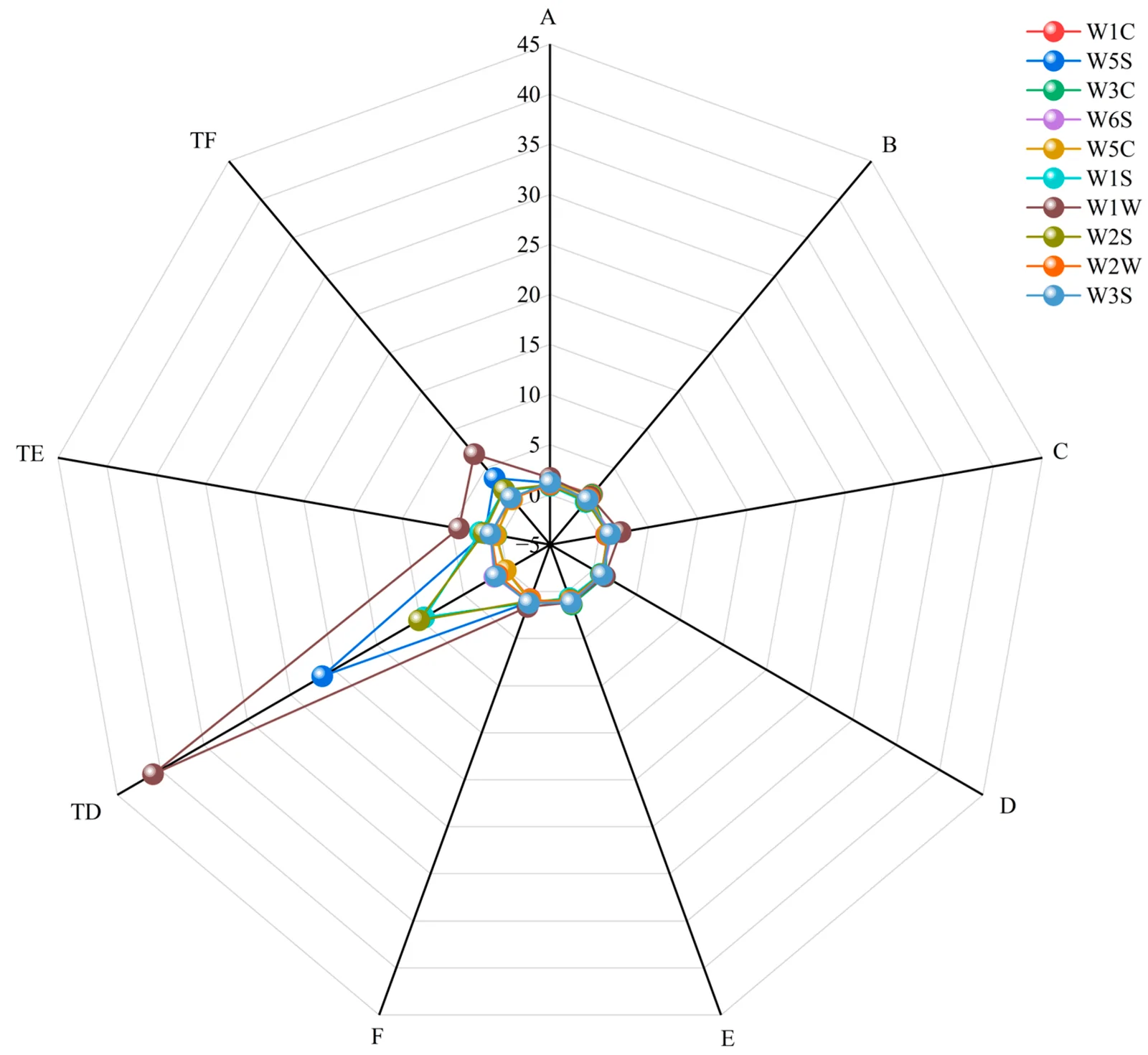
The comparison of different treatment samples of radar map in electronic nose test results.

To further elucidate the variations in volatile flavor compounds among prawn products subjected to different processing methods, the response values of the electronic nose (E-nose) sensors were visualized using a radar chart, as shown in Figure 5. The chart presents the odor fingerprints of nine sample groups (A, B, C, D, E, F, TD, TE, and TF) across ten specific sensors (W1C, W5S, W3C, W6S, W5C, W1S, W1W, W2S, W2W, W3S).

The radar chart revealed a highly polarized distribution pattern. Except for the seasoned cooked prawn (TD), the remaining eight groups (A, B, C, D, E, F, TE, and TF) exhibited notably low response values across all sensors, forming a compact inner core within the center of the radar plot (with sensor responses generally remaining below 10). This indicates that, for the majority of raw, dried, and dried-seasoned products, the concentrations of generated volatile compounds were relatively low under the current testing conditions, or insufficient to trigger extremely high-intensity sensor activations.

In stark contrast, Group TD displayed a distinct and overwhelming outward expansion along specific sensor axes. Detailed analysis showed that the response values for W1W (sulfur compounds) and W5S (nitrogen oxides) spiked dramatically, reaching approximately 40 and 20, respectively. Simultaneously, sensors W1S (alkanes) and W2S (alcohols and some aromatic compounds) also exhibited significant leaps compared to the other groups. This suggests that the seasoning process—typically rich in sugars, spices, and amino acids—when combined with cooked prawn but without subsequent drying (i.e., TD), intensely stimulates the generation of large quantities of sulfur- and nitrogen-containing volatile flavor compounds.

Notably, although the seasoned dried products (TE and TF) did not replicate this extreme outburst, their W1W signals were still noticeably higher than those of the unseasoned samples. This implies that the subsequent dehydration steps (whether freeze-drying or hot-air drying) likely lead to the volatilization or degradation of the highly reactive sulfur/nitrogen compounds generated during the wet-seasoning stage. Alternatively, the drying process may encapsulate these volatile substances within the dense food matrix, thereby inhibiting their release into the headspace during E-nose detection. In summary, the radar chart clearly demonstrates that wet seasoning (TD) acts as a critical “burst point” for the release of volatile sulfides and nitrogen oxides in prawn products, while subsequent drying processes effectively suppress or dissipate this extreme odor profile.

### 3.6. Comparative Analysis of Volatile Organic Compounds

#### 3.6.1. Characterization of Volatile Compound Abundance Based on the Circular Heatmap

**Figure 6:**
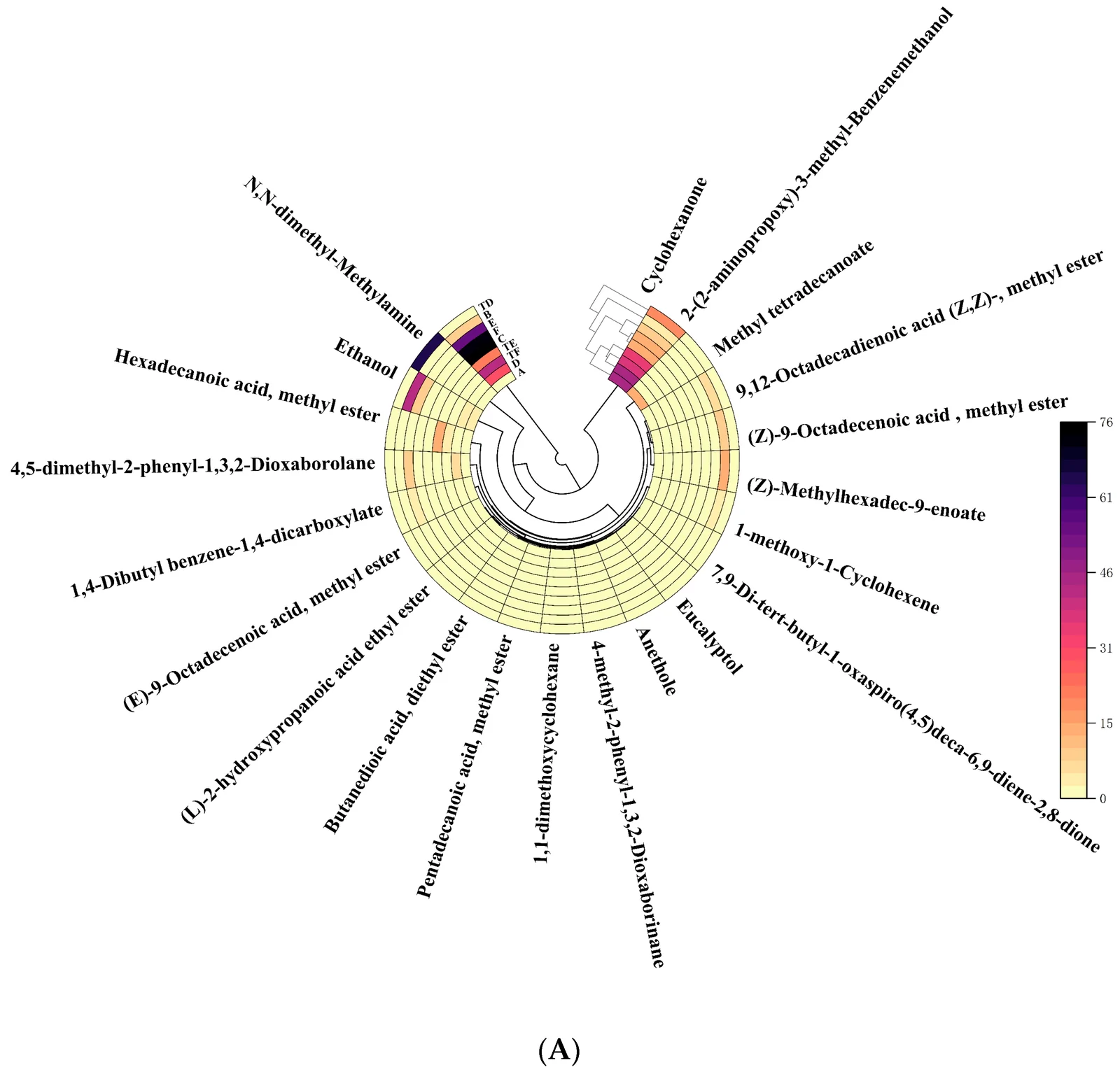

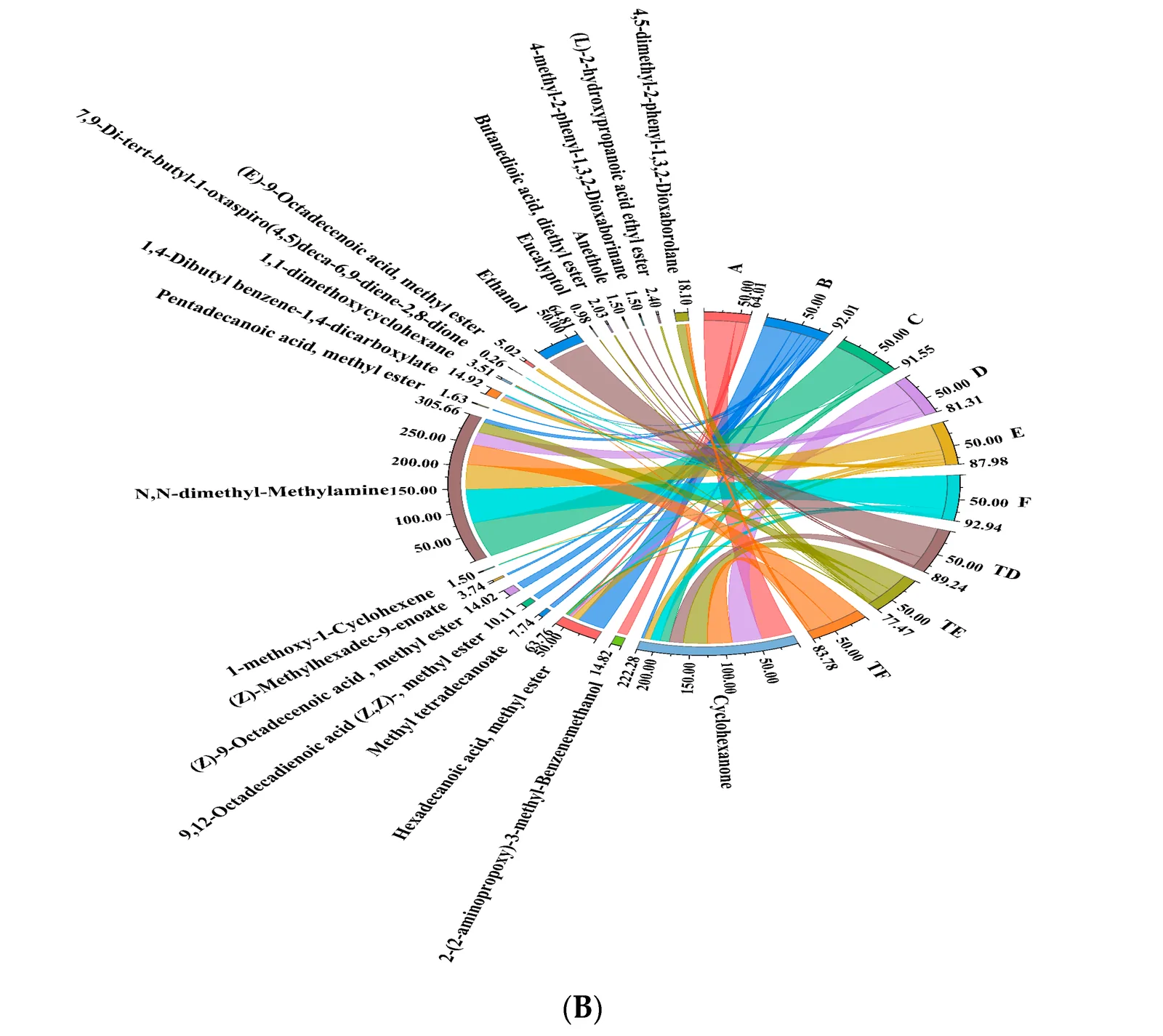
The comparison of circular heat map (**A**) and chord diagram (**B**) of volatile compounds identified by SPME-GC–MS of different treatment samples.

Figure 6A displays the clustering results of the circular heatmap for prawn samples subjected to different processing methods (raw, cooked, dried, and seasoned). By extracting the main compounds with the highest relative abundances in each group, the trajectory of processing-induced changes in the flavor chemical profile of prawn can be clearly delineated. Note: Cyclohexanone was excluded from the compositional analysis as it was artificially introduced as an internal standard (100 µL of a 0.1% *v*/*v* solution).

Raw groups (A, B, C): For A (raw prawn meat), the profile was dominated by 2-(2-aminopropoxy)-3-methyl-benzenemethanol. B (freeze-dried raw prawn meat) exhibited the highest compound diversity, with fatty acid methyl esters (FAMEs) playing a dominant role. The top compounds in terms of abundance included hexadecanoic acid methyl ester, (Z)-9-octadecenoic acid methyl ester, 9,12-octadecadienoic acid (Z,Z)-methyl ester, and methyl tetradecanoate. This suggests that the freeze-drying process maximally preserved the endogenous lipid-derived flavor compounds in raw prawn meat without inducing intense chemical transformations. C (hot-air dried raw prawn meat) showed a dramatic shift, with N, N-dimethyl-methylamine becoming the most prominent non-IS compound. The ranking of endogenous long-chain FAMEs significantly decreased, implying that hot-air drying (thermal processing) promoted the enrichment of amine compounds, potentially derived from amino acid degradation, while also leading to the volatilization or oxidation of some medium-to-high boiling point FAMEs.

Cooked groups (D, E, F): For D (cooked prawn meat), the prominent endogenous compounds were N,N-dimethyl-methylamine, 1,4-dibutyl benzene-1,4-dicarboxylate, and hexadecanoic acid methyl ester. E (freeze-dried cooked prawn meat) displayed a characteristic profile, in which N,N-dimethyl-methylamine ranked first, followed by hexadecanoic acid methyl ester. The combination of cooking and freeze-drying resulted in satisfactory preservation of both amine compounds and endogenous FAMEs. F (hot-air dried cooked prawn meat) exhibited a highly similar profile to group C, with N,N-dimethyl-methylamine as the prominent endogenous compound. This further confirms that the “hot-air drying” operation is a common processing factor driving the evolution of volatile components toward higher amine levels.

Seasoned groups (TD, TE, TF): For TD (seasoned cooked prawn meat), a dramatic compositional shift was observed, with ethanol surging to the top of the abundance list. This indicates that the yellow rice wine (a key seasoning ingredient) played a significant role, and its abundance even overshadowed other flavor compounds generated intrinsically by the prawn meat. For TE (freeze-dried seasoned cooked prawn meat), the prominent non-IS compounds were N,N-dimethyl-methylamine. For TF (hot-air dried seasoned cooked prawn meat), the abundance of N,N-dimethyl-methylamine ranked first among non-IS compounds.

#### 3.6.2. Analysis of the Impact Mechanisms of Processing Techniques on Characteristic Flavor Compounds in Prawn Meat

Combining the clustering heatmap (Figure 6A) with the abundance rankings across the groups, and excluding the influence of the internal standard (cyclohexanone), the following processing mechanisms can be summarized.

Differences in drying methods (freeze-drying vs. hot-air drying): Freeze-drying (B, E, TE) significantly retained the endogenous characteristics of prawn meat, particularly the long-chain fatty acid methyl esters (as seen in group B). The low-temperature and low-oxygen environment greatly suppressed thermally induced degradation reactions. Conversely, hot-air drying (C, F, TF) acted as an intense thermal trigger. Heating disrupted the stability of FAMEs while simultaneously promoting the generation of amine compounds (e.g., N,N-dimethyl-methylamine). This transformation of components serves as the flavor signature of hot-air drying, shifting the profile from lipid-derived esters to nitrogen-containing compounds.

Profound interference of the seasoning process: The seasoning process substantially altered the endogenous flavor metabolite network of the prawn meat. The most prominent characteristic was the high abundance of ethanol in the TD group, attributed directly to the flavoring agent (yellow rice wine).

**Figure 7:**
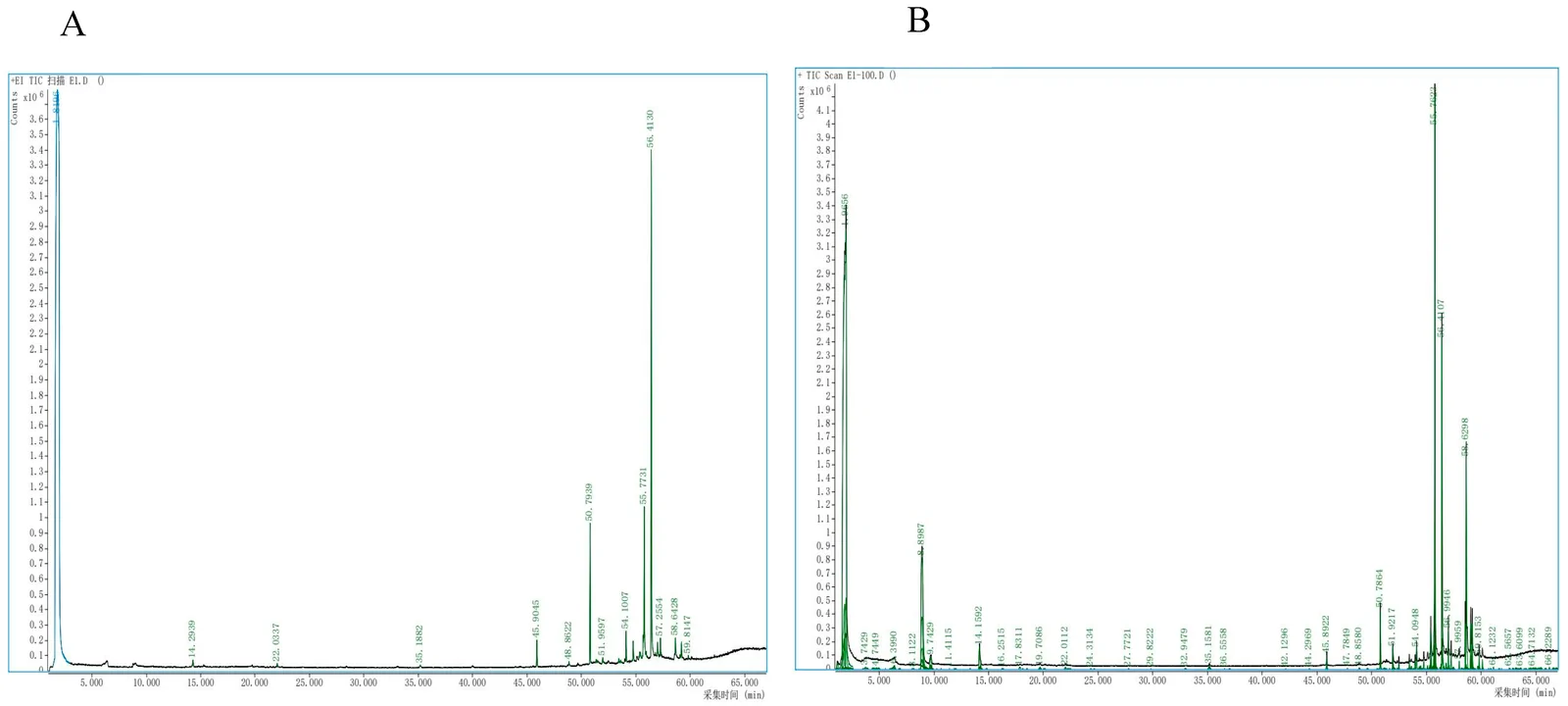
Blank sample (**A**) and spiked sample (**B**) of gas chromatography-mass spectrometry detection spectrum.

The chord diagram in Figure 6B corroborates that the compounds with higher relative abundances identified in the circular heatmap (Figure 6A) exhibit stronger associations with their corresponding sample groups.More detailed interpretation of the data is provided in Figure 7.

## 4. Discussion

### 4.1. Nutrient Remodeling: The Interplay Between Concentration Effects and Thermal Degradation

This study demonstrates that dehydration, irrespective of the drying method employed, serves as an effective means of concentrating nutrients in *Macrobrachium rosenbergii* meat. A significant reduction in moisture content was observed across all dried samples (*p* < 0.05). However, diverging from the findings of Wang et al. [2], who reported no significant change in lipid content following hot-air drying (HAD) of shrimp boiled in 2% (*w*/*v*) NaCl at 100 °C for 2 min, the lipid content in our non-salted, boiled samples increased significantly after HAD. In raw shrimp meat, the HAD group exhibited higher protein and total fatty acid contents (89.27 g/100 g and 4.35 g/100 g, respectively) compared to the vacuum freeze-drying (VFD) group. Conversely, in cooked shrimp meat, VFD markedly outperformed HAD in preserving heat-labile amino acids, such as lysine and arginine, as well as polyunsaturated fatty acids (PUFAs). This observation aligns with the findings of Liu et al. [9].

Furthermore, our study highlights the unique behavior of cystine, which was identified exclusively in the unseasoned cooked groups (D, E, F) and was entirely absent in all other samples. This suggests that in thermally processed, unseasoned shrimp, heat-induced protein denaturation liberates substantial amounts of free cysteine. Given that free cysteine is highly unstable, its thiol (-SH) side chains are prone to oxidative condensation under heating and aerobic conditions, forming disulfide bonds (-S-S-) that dimerize to yield cystine. Consequently, cystine was identified as a prominent product of heat-induced protein oxidation and denaturation in the unseasoned cooked groups. In raw shrimp and its products, the absence of cystine is primarily attributable to its marked instability. The literature indicates that free L-cysteine is readily oxidized upon exposure to air, converting to L-cystine; simultaneously, cystine itself can be reduced, leading to disulfide bond cleavage and regeneration of cysteine. In the complex enzymatic and uncontrolled redox environment of raw samples, cysteine and cystine exist in a dynamic equilibrium of rapid interconversion. Coupled with environmental interferences during extraction and detection, this precludes the stable detection of cystine in raw samples [30,31]. In the seasoned groups, the interplay of complex Maillard reactions and reducing conditions further contributed to the disappearance of cystine [32]. Therefore, while HAD imparts a stronger physical concentration effect on raw materials, VFD is indispensable for preserving the delicate nutritional integrity of cooked shrimp meat and preventing the severe depletion of heat-labile amino acids driven by Maillard reactions.

### 4.2. Mechanistic Divergence in Volatile Flavor Formation

The most prominent flavor differences between VFD and HAD are driven by fundamentally distinct chemical mechanisms: physical concentration versus thermochemical induction. Electronic nose and GC-MS analyses revealed that VFD preserves the endogenous volatile signature of the shrimp meat, characterized by long-chain fatty acid methyl esters (FAMEs) in the raw material (Group B) and a balanced amine–ester profile in the cooked products. This is consistent with previous research indicating that, compared to traditional boiling, VFD, akin to vacuum low-temperature cooking, provides a gentler processing method for shrimp, resulting in more tightly aligned myofibrils, improved water-holding capacity, more palatable textural properties, and higher levels of umami amino acids, thereby highlighting its unique advantage as a rich source of savory compounds [33].

In contrast, HAD acts as a potent thermally triggered engine. Our results demonstrate a sharp transition in the flavor profile of HAD groups from lipid-derived FAMEs to nitrogen-containing compounds such as N,N-dimethylmethanamine. This transformation is ascribed to the intensification of Maillard reactions and Strecker degradation at elevated temperatures, which consume reducing sugars and amino acids to generate pyrazines, amines, and heterocyclic compounds [34]. Liang et al. provided further mechanistic insights by constructing a reaction model simulating 85 °C hot-air drying. They demonstrated that lysine (Lys) and arginine (Arg) serve as the primary amino acid precursors for the formation of 13 aroma-active pyrazines (e.g., 2-ethyl-3,6-dimethylpyrazine, 2,5-dimethylpyrazine) and trimethylamine, while glucose acts as the predominant carbonyl precursor. Our detection of N,N-dimethylmethanamine and the disappearance of cystine in the HAD groups are in full agreement with their conclusion that high temperatures promote the thermal degradation of specific amino acids, such as Lys and Arg, to generate nitrogenous compounds [35].

### 4.3. Reconstruction of Non-Volatile Taste Profiles: Concentration, Generation, and Masking

Electronic tongue analysis indicated that drying intensified the saltiness, umami, and richness across all samples, a direct consequence of concentrating water-soluble taste-active compounds [36]. However, the interaction between processing stage and drying method yielded distinctly different taste outcomes. Group B (raw, freeze-dried shrimp) displayed significantly higher values for Saltiness, Astringency Aftertaste (After-A), Bitterness Aftertaste (After-B), and Richness compared to other samples, while exhibiting lower sweetness and bitterness. These findings are largely consistent with those of Liang et al. [35]. When applied to pre-cooked raw materials, the two drying methods led to divergent concentration and/or generation pathways for taste compounds—freeze-drying tended to preserve or enhance umami-related nucleotides [7], whereas hot-air drying accentuated the sensory experience of saltiness and kokumi-like mouthfulness [37]. The seasoning process superimposed a strong salty-acidic flavor profile, which masked the inherent delicate sweetness and umami of the shrimp meat, a phenomenon known as cross-modal taste interaction. Interestingly, even after seasoning, VFD (Group TE) remained superior to HAD (Group TF) in preserving umami and richness. This observation can be explained by the “embedding” effect of freeze-drying, which prevents the migration of taste substances to the surface; conversely, HAD promotes the surface migration of salts and sugars (crusting effect), potentially leading to uneven taste distribution and greater loss of umami precursors [36].

### 4.4. Implications for Process Optimization and Industrial Applications

Based on the comprehensive analysis of nutritional retention, flavor formation, and taste attributes, this study provides clear guidance for industrial production. For manufacturers aiming to produce high-end ready-to-eat dried shrimp floss with superior nutritional quality and a natural seafood flavor, the cooking “followed by VFD” (Cooking → VFD) process route is recommended, as it maximizes the retention of heat-labile nutrients (such as PUFAs and sulfur-containing amino acids) while maintaining the inherent taste balance [3]. Furthermore, our findings suggest that HAD may be more suitable for developing products that emphasize intense roasted aromas and a highly concentrated taste profile, albeit with lower retention of sensitive nutrients [37]. Nevertheless, the thermally induced oxidative pathways during HAD must be carefully controlled. As highlighted by Gao et al.in their study on shrimp paste post-ripening, light exposure (a form of thermal/photo-oxidative stress) promotes oxidative degradation of fatty acids, generating a variety of flavor compounds (aldehydes, ketones, esters) [33]. Similarly, the intense heat during our HAD process accelerated lipid autoxidation, which not only produced roasted aromas but also consumed essential PUFAs [38]. Therefore, for HAD applications, future strategies could involve the use of natural antioxidants or the optimization of drying temperature profiles to mitigate excessive fatty acid degradation while still achieving the desired roasted sensory characteristics. This study establishes a theoretical framework for selecting appropriate drying technologies based on the targeted product positioning and sensory requirements.

## 5. Conclusions

This study comprehensively elucidated the quality evolution of giant freshwater prawn meat subjected to VFD and HAD across raw, cooked, and seasoned processing stages. The principal conclusions are drawn as follows:

1. Nutrient retention is governed by interactive effects of drying method and pre-treatment. Drying significantly enhanced nutritional density via moisture removal. For raw prawn, HAD induced a stronger apparent concentration of proteins and fatty acids due to more extensive dehydration. However, in cooked prawn, VFD was markedly superior in preserving amino acids and PUFAs, as thermal degradation and Maillard participation during HAD led to substantial losses. The “cooking prior to VFD” route is therefore identified as the optimal strategy for maximizing nutritional quality.

2. Volatile flavor formation follows distinct mechanistic pathways. VFD predominantly acted as a physical concentrator, retaining the intrinsic volatile signature of prawn, including lipid-derived FAMEs. In contrast, HAD functioned as a thermochemical engine, driving the transformation of esters into nitrogen-rich compounds such as N,N-dimethylmethanamine and generating roasted markers, which fundamentally reshaped the aroma profile. Seasoning was confirmed as the primary driver of flavor divergence, with drying method playing a secondary yet significant role.

3. Non-volatile taste profiles are reconfigured through concentration, generation, and masking effects. Drying universally intensified saltiness, umami, and richness via tastant concentration. VFD-cooked prawn achieved the optimal synergy between cooking-induced umami generation and freeze-drying preservation, whereas HAD tended to intensify astringency, saltiness, and richness. Seasoning exerted a strong salty-acidic masking effect.

4. Processing-induced cystine behavior provides a novel chemical marker. Cystine was exclusively identified in unseasoned cooked groups, indicating heat-induced cysteine oxidation and disulfide formation. Its complete absence in raw and seasoned samples reflects the instability of the cysteine–cystine redox equilibrium and the interfering effects of Maillard-derived reducing conditions, offering new insights into protein oxidation dynamics during processing.

5. Industrial implications are clearly delineated. For high-end products emphasizing authentic flavor and nutritional integrity, VFD is the preferred technology. For products targeting intense roasted notes and concentrated mouthfeel, HAD remains advantageous, provided that oxidative degradation is carefully controlled via antioxidant strategies or optimized temperature profiles.

In summary, this study establishes a scientific and theoretical foundation for rational selection of drying technologies and precise flavor regulation in dried aquatic products, bridging the gap between processing parameters and final quality attributes.

## Figures and Tables

**Table 1:** German PEN3 electronic nose sensor sensitive substances.

Sensor No.	Sensor Code	Sensitive Substances
1	W1C	Aromatic compounds
2	W5S	Nitrogen oxides
3	W3C	Ammonia, aromatic molecules
4	W6S	Hydrides
5	W5C	Alkenes, aromatic compounds, polar molecules
6	W1S	Alkanes
7	W1W	Sulfur compounds
8	W2S	Alcohols, some aromatic compounds
9	W2W	Aromatic compounds, organic sulfur compounds
10	W3S	Alkanes and aliphatic compounds

**Table 2:** The matching information between the sensor and the taste value.

Sensor	Corresponding Taste	Taste Information	
		Initial Taste	Aftertaste
C00	Sourness-bitterness	Bitterness	Aftertaste-B
AE1	Astringency	Astringency	Aftertaste-A
CA0	Sourness	Sourness	
CT0	Saltness	Saltness	
AAE	Umami	Umami	Richness
GL1	Sweetness	Sweetness	

**Table 3:** Proximate composition (dry matter basis) of prawn meat under different processing treatments (g/100 g).

	A	B	C	D	E	F	TD	TE	TF
	Mean ± RSD%								
protein g/100 g	86.32 ± 1.81 ^a^	83.75 ± 0.92 ^b^	89.27 ± 0.10 ^c^	85.29 ± 1.12 ^a^	85.91 ± 0.25 ^a^	85.61 ± 0.58 ^a^	75.08 ± 0.75 ^d^	71.18 ± 0.12 ^e^	74.76 ± 0.09 ^d^
fat g/100 g	3.20 ± 0.31 ^a^	4.26 ± 0.12 ^b^	4.65 ± 0.03 ^b^	8.54 ± 0.20 ^c^	8.79 ± 0.29 ^c^	8.98 ± 0.13 ^c^	11.18 ± 0.42 ^d^	6.70 ± 0.61 ^e^	7.00 ± 0.10 ^e^
carbohydrate g/100 g	4.10 ± 0.44 ^a^	6.13 ± 0.86 ^b^	0.08 ± 0.01 ^c^	1.66 ± 0.17 ^c^	0.65 ± 0.06 ^c^	0.75 ± 0.05 ^c^	6.25 ± 0.10 ^b^	14.72 ± 0.57 ^d^	10.60 ± 0.11 ^e^
ash g/100 g	6.38 ± 0.13 ^a^	5.86 ± 0.06 ^b^	6.00 ± 0.03 ^c^	4.51 ± 0.09 ^d^	4.65 ± 0.04 ^d^	4.66 ± 0.03 ^d^	7.50 ± 0.09 ^e^	7.38 ± 0.12 ^e^	7.64 ± 0.10 ^f^
sodium mg/100 g	0.30 ± 0.00 ^a^	0.37 ± 0.00 ^a^	0.40 ± 0.01 ^a^	0.35 ± 0.02 ^a^	0.33 ± 0.01 ^a^	0.33 ± 0.01 ^a^	1.63 ± 0.05 ^b^	1.99 ± 0.19 ^c^	1.84 ± 0.04 ^d^
total fatty acids g/100 g	2.97 ± 0.05 ^a^	4.08 ± 0.16 ^b^	4.35 ± 0.09 ^c^	7.43 ± 0.15 ^d^	7.75 ± 0.25 ^e^	7.79 ± 0.11 ^e^	8.44 ± 0.23 ^f^	5.89 ± 0.26 ^g^	5.40 ± 0.07 ^h^
total amino acid g/100 g	62.32 ± 2.47 ^a^	85.88 ± 5.31 ^b^	82.67 ± 0.42 ^b^	83.73 ± 1.58 ^b^	91.10 ± 1.84 ^c^	90.31 ± 3.16 ^c^	70.97 ± 1.38 ^d^	70.97 ± 3.75 ^d^	71.23 ± 1.08 ^d^
water content g/100 g	76.56 ± 0.30 ^a^	21.93 ± 0.09 ^b^	9.72 ± 0.16 ^c^	73.67 ± 0.11 ^d^	5.36 ± 0.09 ^e^	11.64 ± 0.12 ^f^	70.79 ± 0.11 ^g^	12.96 ± 1.23 ^h^	13.97 ± 0.11 ^i^

Different superscript letters indicate significant differences (*p* < 0.05). A (raw prawn meat), B (VFD raw prawn meat), C (HAD raw prawn meat), D (cooked prawn meat without seasoning), E (VFD cooked prawn meat without seasoning), F (HAD cooked prawn meat without seasoning), TD (cooked prawn meat with seasoning), TE (VFD cooked prawn meat with seasoning), TF (HAD cooked prawn meat with seasoning).

**Table 4:** Amino acid composition (dry matter basis) of prawn meat under different processing treatments (g/100 g).

	A	B	C	D	E	F	TD	TE	TF
	Mean ± RSD%								
Asp	6.44 ± 0.35 ^a^	8.97 ± 0.46 ^b^	8.33 ± 0.08 ^c^	8.85 ± 0.23 ^b^	9.82 ± 0.19 ^d^	9.14 ± 0.49 ^b^	7.76 ± 0.11 ^e^	7.61 ± 0.13 ^e^	7.51 ± 0.13 ^e^
Thr	2.30 ± 0.10 ^a^	3.14 ± 0.12 ^b^	2.98 ± 0.07 ^bf^	3.71 ± 0.26 ^c^	4.38 ± 0.22 ^d^	4.03 ± 0.43 ^e^	2.81 ± 0.03 ^bf^	2.69 ± 0.15 ^f^	2.71 ± 0.05 ^f^
Ser	2.10 ± 0.07 ^a^	2.85 ± 0.04 ^b^	2.55 ± 0.15 ^c^	3.41 ± 0.07 ^d^	3.76 ± 0.08 ^e^	3.61 ± 0.20 ^de^	2.57 ± 0.09 ^c^	2.53 ± 0.30 ^c^	2.43 ± 0.09 ^c^
Glu	10.76 ± 0.82 ^a^	13.06 ± 0.92 ^bc^	12.68 ± 0.12 ^bcd^	12.67 ± 0.39 ^bcd^	13.72 ± 0.38 ^c^	13.64 ± 0.72 ^c^	12.01 ± 0.26 ^bd^	11.58 ± 0.56 ^d^	11.80 ± 0.19 ^d^
Gly	3.28 ± 0.34 ^a^	4.28 ± 0.49 ^ab^	4.37 ± 0.06 ^ab^	3.79 ± 0.07 ^a^	4.15 ± 0.15 ^ab^	5.16 ± 0.17 ^b^	3.59 ± 0.14 ^a^	3.49 ± 0.18 ^a^	3.40 ± 0.05 ^a^
Ala	4.53 ± 0.14 ^ab^	4.63 ± 0.38 ^ab^	4.90 ± 0.13 ^b^	4.45 ± 0.08 ^ac^	4.68 ± 0.13 ^a^	4.79 ± 0.36 ^ab^	4.40 ± 0.22 ^ac^	4.11 ± 0.08 ^c^	4.03 ± 0.06 ^cd^
Cys	0.00 ± 0.00 ^a^	0.00 ± 0.00 ^a^	0.00 ± 0.00 ^a^	0.34 ± 0.12 ^b^	0.35 ± 0.03 ^b^	0.44 ± 0.11 ^c^	0.00 ± 0.00 ^a^	0.00 ± 0.00 ^a^	0.00 ± 0.00 ^a^
Val	3.35 ± 0.07 ^a^	4.17 ± 0.25 ^b^	4.10 ± 0.07 ^b^	4.60 ± 0.07 ^c^	4.97 ± 0.13 ^d^	4.76 ± 0.13 ^cd^	4.05 ± 0.15 ^b^	3.49 ± 0.43 ^a^	3.68 ± 0.07 ^a^
Met	1.82 ± 0.04 ^a^	2.86 ± 0.20 ^b^	2.71 ± 0.03 ^bc^	2.55 ± 0.21 ^c^	3.17 ± 0.07 ^d^	3.10 ± 0.04 ^d^	1.59 ± 0.21 ^e^	2.08 ± 0.25 ^f^	2.11 ± 0.03 ^f^
Ile	3.64 ± 0.05 ^a^	4.45 ± 0.24 ^b^	4.22 ± 0.04 ^bcd^	3.88 ± 0.06 ^ac^	4.12 ± 0.13 ^bcd^	3.95 ± 0.19 ^acd^	4.28 ± 0.12 ^bc^	3.70 ± 0.44 ^a^	3.66 ± 0.05 ^a^
Leu	5.10 ± 0.09 ^a^	7.58 ± 0.53 ^b^	7.46 ± 0.04 ^b^	6.21 ± 0.12 ^c^	6.58 ± 0.18 ^cd^	6.41 ± 0.21 ^cd^	7.05 ± 0.13 ^db^	6.40 ± 0.76 ^cd^	6.66 ± 0.13 ^cd^
Tyr	4.00 ± 0.16 ^a^	4.24 ± 0.12 ^a^	4.02 ± 0.19 ^a^	3.24 ± 0.26 ^bc^	3.36 ± 0.09 ^b^	3.08 ± 0.21 ^bc^	2.71 ± 0.29 ^c^	2.83 ± 0.61 ^bc^	3.16 ± 0.06 ^bc^
Phe	2.90 ± 0.20 ^a^	3.83 ± 0.23 ^b^	3.72 ± 0.02 ^b^	3.53 ± 0.15 ^b^	3.87 ± 0.09 ^bc^	3.60 ± 0.22 ^bc^	3.57 ± 0.04 ^bc^	3.25 ± 0.46 ^c^	3.43 ± 0.05 ^bc^
Lys	4.69 ± 0.24 ^a^	7.67 ± 0.23 ^b^	7.12 ± 0.06 ^c^	8.27 ± 0.27 ^d^	9.02 ± 0.16 ^e^	9.02 ± 0.36 ^e^	6.57 ± 0.14 ^f^	6.41 ± 0.13 ^fg^	6.11 ± 0.11 ^g^
His	1.78 ± 0.09 ^a^	2.84 ± 0.20 ^b^	2.73 ± 0.02 ^bc^	2.72 ± 0.11 ^bc^	2.81 ± 0.07 ^b^	2.76 ± 0.10 ^bc^	2.62 ± 0.04 ^bc^	2.25 ± 0.39 ^d^	2.44 ± 0.04 ^cd^
Arg	3.62 ± 0.10 ^a^	8.43 ± 0.78 ^bc^	8.21 ± 0.05 ^b^	8.41 ± 0.34 ^bc^	9.06 ± 0.21 ^c^	9.14 ± 0.65 ^c^	3.31 ± 0.35 ^a^	6.12 ± 0.44 ^d^	6.22 ± 0.09 ^d^
Pro	2.00 ± 0.21 ^a^	2.89 ± 0.15 ^bc^	2.54 ± 0.06 ^abc^	3.10 ± 0.18 ^bcd^	3.27 ± 0.11 ^cd^	3.71 ± 0.92 ^d^	2.11 ± 0.08 ^a^	2.41 ± 0.57 ^ab^	1.89 ± 0.07 ^a^
Trp	10.25 ± 0.03 ^a^	12.16 ± 0.04 ^b^	10.02 ± 0.09 ^c^	9.52 ± 0.06 ^d^	9.41 ± 0.14 ^e^	10.66 ± 0.07 ^f^	8.60 ± 0.05 ^g^	9.82 ± 0.15 ^c^	9.11 ± 0.07 ^h^

Different superscript letters indicate significant differences (*p* < 0.05). A (raw prawn meat), B (VFD raw prawn meat), C (HAD raw prawn meat), D (cooked prawn meat without seasoning), E (VFD cooked prawn meat without seasoning), F (HAD cooked prawn meat without seasoning), TD (cooked prawn meat with seasoning), TE (VFD cooked prawn meat with seasoning), TF (HAD cooked prawn meat with seasoning).

**Table 5:** Fatty acid composition (dry matter basis) of prawn meat under different processing treatments (g/100 g).

	A	B	C	D	E	F	TD	TE	TF
	Mean ± RSD%								
DHA	0.24 ± 0.01 ^a^	0.29 ± 0.01 ^b^	0.31 ± 0.01 ^b^	0.36 ± 0.01 ^ce^	0.38 ± 0.01 ^c^	0.38 ± 0.00 ^c^	0.48 ± 0.05 ^d^	0.38 ± 0.04 ^c^	0.34 ± 0.00 ^e^
EPA	0.42 ± 0.01 ^a^	0.43 ± 0.03 ^ab^	0.46 ± 0.01 ^abc^	0.46 ± 0.00 ^abcd^	0.48 ± 0.01 ^cd^	0.48 ± 0.01 ^bcd^	0.51 ± 0.04 ^d^	0.47 ± 0.03 ^bcd^	0.42 ± 0.01 ^a^
C18:0	0.50 ± 0.03 ^a^	0.48 ± 0.02 ^a^	0.52 ± 0.01 ^a^	0.83 ± 0.02 ^b^	0.85 ± 0.02 ^b^	0.85 ± 0.01 ^b^	0.95 ± 0.08 ^c^	0.61 ± 0.01 ^d^	0.59 ± 0.01 ^d^
C16:0	0.84 ± 0.04 ^a^	0.93 ± 0.01 ^b^	0.98 ± 0.02 ^b^	1.70 ± 0.05 ^c^	1.78 ± 0.06 ^d^	1.80 ± 0.02 ^d^	2.04 ± 0.04 ^e^	1.42 ± 0.08 ^f^	1.26 ± 0.02 ^g^
C17:0	0.035 ± 0.006 ^a^	0.045 ± 0.002 ^ab^	0.047 ± 0.001 ^b^	0.058 ± 0.003 ^c^	0.065 ± 0.002 ^c^	0.066 ± 0.002 ^c^	0.063 ± 0.015 ^c^	0.045 ± 0.001 ^ab^	0.045 ± 0.001 ^ab^
C14:0	0.029 ± 0.003 ^a^	0.036 ± 0.002 ^a^	0.039 ± 0.001 ^a^	0.170 ± 0.008 ^b^	0.166 ± 0.006 ^b^	0.172 ± 0.004 ^b^	0.214 ± 0.026 ^c^	0.105 ± 0.008 ^d^	0.107 ± 0.003 ^d^
C15:0	0.00 ± 0.00 ^a^	0.00 ± 0.00 ^a^	0.00 ± 0.00 ^a^	0.014 ± 0.016 ^b^	0.036 ± 0.003 ^c^	0.039 ± 0.001 ^c^	0.00 ± 0.00 ^a^	0.00 ± 0.00 ^a^	0.00 ± 0.00 ^a^
C18:3	0.065 ± 0.006 ^a^	0.066 ± 0.004 ^a^	0.070 ± 0.003 ^a^	0.129 ± 0.003 ^b^	0.135 ± 0.002 ^b^	0.139 ± 0.002 ^b^	0.167 ± 0.008 ^c^	0.114 ± 0.014 ^d^	0.098 ± 0.001 ^e^
C20:2	0.046 ± 0.002 ^a^	0.058 ± 0.002 ^b^	0.060 ± 0.001 ^b^	0.116 ± 0.006 ^c^	0.129 ± 0.003 ^d^	0.124 ± 0.004 ^e^	0.151 ± 0.004 ^f^	0.094 ± 0.005 ^g^	0.085 ± 0.000 ^h^
C18:2	0.786 ± 0.033 ^a^	0.764 ± 0.027 ^a^	0.803 ± 0.018 ^a^	1.408 ± 0.033 ^b^	1.476 ± 0.048 ^c^	1.499 ± 0.020 ^c^	1.669 ± 0.027 ^d^	1.143 ± 0.043 ^e^	1.065 ± 0.017 ^f^
C20:1	0.018 ± 0.001 ^a^	0.014 ± 0.001 ^b^	0.016 ± 0.001 ^ab^	0.053 ± 0.004 ^c^	0.056 ± 0.002 ^cd^	0.054 ± 0.002 ^c^	0.059 ± 0.002 ^d^	0.035 ± 0.002 ^e^	0.032 ± 0.001 ^e^
C20:4	0.120 ± 0.006 ^a^	0.137 ± 0.008 ^b^	0.150 ± 0.002 ^cd^	0.134 ± 0.002 ^b^	0.152 ± 0.003 ^cd^	0.145 ± 0.004 ^c^	0.155 ± 0.008 ^d^	0.131 ± 0.002 ^b^	0.132 ± 0.002 ^b^
C18:1	0.761 ± 0.035 ^a^	0.790 ± 0.041 ^a^	0.851 ± 0.016 ^a^	1.718 ± 0.051 ^b^	1.76 ± 0.06 ^b^	1.746 ± 0.027 ^b^	1.79 ± 0.03 ^b^	1.33 ± 0.16 ^c^	1.11 ± 0.01 ^d^
C16:1	0.091 ± 0.007 ^a^	0.058 ± 0.004 ^b^	0.058 ± 0.001 ^b^	0.303 ± 0.004 ^c^	0.30 ± 0.01 ^c^	0.302 ± 0.006 ^c^	0.19 ± 0.01 ^d^	0.14 ± 0.03 ^e^	0.11 ± 0.00 ^a^

Different superscript letters indicate significant differences (*p* < 0.05). A (raw prawn meat), B (VFD raw prawn meat), C (HAD raw prawn meat), D (cooked prawn meat without seasoning), E (VFD cooked prawn meat without seasoning), F (HAD cooked prawn meat without seasoning), TD (cooked prawn meat with seasoning), TE (VFD cooked prawn meat with seasoning), TF (HAD cooked prawn meat with seasoning).

## Data Availability

The original contributions presented in the study are included in the article. Further inquiries can be directed to the corresponding author.
